# Mucoadhesive Dendrons Conjugated to Mesoporous Silica Nanoparticles as a Drug Delivery Approach for Orally Administered Biopharmaceuticals

**DOI:** 10.1021/acsami.2c16502

**Published:** 2023-02-07

**Authors:** Matteo Tollemeto, Zheng Huang, Jørn B. Christensen, Hanne Mørck Nielsen, Stine Rønholt

**Affiliations:** † Department of Chemistry, University of Copenhagen, Thovaldsensvej 40, DK-1871 Frederiksberg, Denmark; ‡ Center for Biopharmaceuticals and Biobarriers in Drug Delivery (BioDelivery), Department of Pharmacy, Faculty of Health and Medical Sciences, University of Copenhagen, Universitetsparken 2, 2100 Copenhagen Ø, Denmark

**Keywords:** novel synthesis, mucus interaction, nanomedicine, gastrointestinal tract, biobarriers, cationic polymers

## Abstract

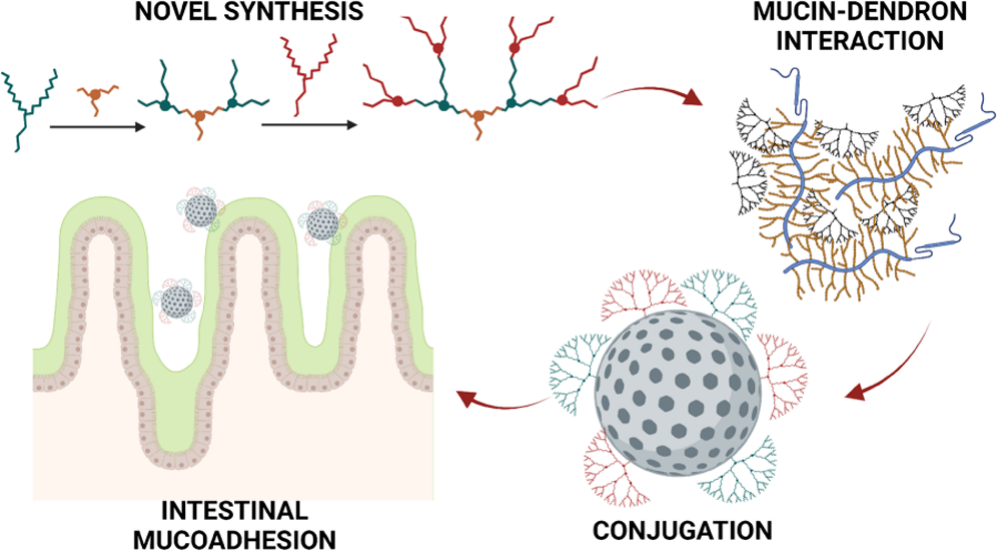

Biological drugs are increasingly important for patients and industry due to their application in the treatment of common and potentially life-threatening diseases such as diabetes, cancer, and obesity. While most marketed biopharmaceuticals today are injectables, the potential of mucoadhesive delivery systems based on dendron-coated mesoporous silica nanoparticles for oral delivery of biological drugs is explored in this project. We hypothesize that specifically designed dendrons can be employed as mucoadhesive excipients and used to decorate the surface of nanoparticles with properties to embed a drug molecule. We initially tested a novel synthesis method for the preparation of dendrons, which was successfully validated by the chemical characterization of the compounds. The interaction between dendrons and mucin was studied through isothermal titration calorimetry and quartz crystal microbalance with dissipation monitoring and proved to be spontaneous and thermodynamically favorable. Dendrons were conjugated onto 244.4 nm mesoporous silica nanoparticles and characterized for chemical composition, size, and surface charge, which all showed a successful conjugation. Finally, dynamic light scattering was used to study the interaction between nanoparticles and porcine gastric mucin, whereas the interaction between nanoparticles and porcine intestinal mucus was characterized by rheological measurements. This study shows a deeper biophysical understanding of the interaction between nanoparticles and mucin or native porcine intestinal mucus, further leveraging the current understanding of how dendrons can be used as excipients to interact with mucin. This will provide knowledge for the potential development of a new generation of mucoadhesive nanoformulations for the oral delivery of biopharmaceuticals.

## Introduction

Despite oral administration being the preferred delivery route of medicines, successful oral delivery of biological drugs is challenged by drug stability issues in the gastrointestinal tract (GIT). Thus, many drugs are precluded from this administration route, necessitating administration by injection.^1^ One prominent example is insulin, which is usually administered by subcutaneous injection. By oral delivery, the signal transduction pathway of insulin could be more precisely replicated, and with optimal formulations, this would lead to a decreased risk of triggering hyperinsulinemia.^2,3^ Furthermore, oral delivery of insulin, being noninvasive, would increase patient convenience, reduce complications by invasive administration,^2^ and could reduce the environmental impact with regard to waste disposal associated with injections.^4,5^ However, oral delivery of insulin still remains elusive in drug delivery, as there are a number of barriers and challenges preventing full implementation for oral administration to be the preferred administration route.^4,6^ Upon administration, insulin must be absorbed as a structurally intact molecule to remain fully effective.^7^ Consequently, oral administration of insulin faces many difficulties in the GIT, which can cause reduced absorption from the small intestine and thus limited bioavailability.^7^ The low pH in the stomach is the first barrier opposed to oral delivery of insulin, as it readily degrades the protein structure.^8^ Additionally, many of the digestive proteolytic enzymes are found in the stomach and intestines, and these are responsible for protein breakdown, including insulin, reducing its bioavailability.^3,4^ The mucus layer, found on the surface of epithelial cells, works as a protective sheet against bacteria and viruses due to, amongst other properties, its high viscosity. However, it also acts as a barrier for drug diffusion toward epithelium.^3^ The epithelial layer, formed of tightly interconnected cells, possesses yet another limitation for sufficient bioavailability of therapeutic peptides and proteins due to their large molecular size.^7,3,4,8^

One strategy to overcome these challenges is to use nanoparticles, as their increased specific surface area allows for a higher degree of interaction with the mucus layer and thus a longer residence time in the GIT compared to that of traditional tablets.^9^ Further, nanoparticles can be fine-tuned and engineered according to the desired needs. For example, they can be designed to be mucoadhesive or to respond to some sort of stimuli.^10^ An example is reported by Cheng et al.,^10^ who showed that insulin loaded in poly(butyl cyanoacrylate) nanoparticles coated with chitosan or alginate increased the mucoadhesion and thus prolonged the residence time in the small intestine upon oral administration to rats when compared to their noncoated nanoparticle counterpart.

In the last few years, several reports have shown how mesoporous silica nanoparticles (MeSiNP) constitute a successful oral carrier for poorly soluble drugs. This is due to their ability to withstand harsh conditions in the gastrointestinal environment and high loading capacity, which protects the encapsulated drug from degradation, resulting in increased bioavailability.^11−16^ A report from Desai et al.^16^ describes how orally administered MeSiNP, when derivatized with poly(ethylene glycol) and poly(ethylene imine) coatings, are able to permeate the intestinal mucus barrier for successful delivery of an anticancer drug. A major limitation of MeSiNP is, however, to protect hydrophilic drugs from solubilization when embedded inside the pores and to selectively release the drug in close proximity to the mucus and epithelial barrier in the gut.^15,17^

Preliminary studies reporting on dendrimers, highly branched polymeric molecules, have shown mucoadhesion of both thiolated and amine-terminated polyamidoamine (PAMAM) dendrimers.^18,19^ Thus, there is a strong rationale for further studies of their interactions with mucus. Dendrons are branched polymeric structures that are closely related to dendrimers. They differ from dendrimers by possessing only a single focal point but are similar in that they can be functionalized with different terminal groups either at the chain ends or at the focal point.^20^ The possibility of fine-tuning the dendron’s outer core with different functional groups provides a window of opportunities to enhance and exploit structural effects on mucoadhesion. Functionalization of the dendrons to the particles can further be designed to respond to external stimuli such as pH changes.^21^ In this project, we study bare MeSiNP and generation 1 (G1) dendron-conjugated MeSiNP by orthogonal methods to provide a first insight into the potential application of tailor-made dendrons as decoration molecules in oral nanoparticulate drug delivery systems.

In literature, two commonly used methods for the conjugation of dendrimers or dendrons onto the surface of MeSiNP are reported; conjugation of separately synthesized dendrimers onto the MeSiNP or growing dendrons directly on the surface of MeSiNP, respectively. The first step in the conjugation method is the functionalization of the MeSiNP with 3-isocyanatopropyltriethoxysilane. The 3-isocyanatopropyl-terminated MeSiNP then react with the amino groups on the PAMAM dendrimer.^22^ One major problem with the conjugation of higher generations of PAMAM dendrimers is their possible toxicity due to a large number of positively charged amine groups on the surface.^23^ Additionally, the steric hindrance around the nanoparticles could affect and reduce the loading and/or release of biological drugs from the particles if not properly triggered. To reduce and avoid toxic response due to a large number of charged amine groups while still preserving mucoadhesion, coating the nanoparticles with dendrons instead could be a relevant approach. Currently, MeSiNP conjugated with dendrons are synthesized using the sol–gel method.^24^ After the nanoparticles are synthesized, they are dispersed in ethanol and (3-aminopropyl)triethoxysilane to form a G0_MeSiNP. The dendrons on the nanoparticles are then further grown to higher generations by following a Michael addition reaction where methyl acrylate is first added to the mixture, and then after washing, the coated nanoparticles are reacted with ethylenediamine to yield a G1_MeSiNP.^19^ The limitation of this latter technique is that while it might be used to grow first-generation dendrons, creating higher generations may be difficult due to the risk of several side reactions taking place.^25^ Therefore, we suggest a new synthetic route for dendron synthesis and conjugation to MeSiNP.

In this paper, we present dendron-functionalized MeSiNP, prepared by a novel synthesis route, as new mucoadhesive nanoparticles that can be used for drug delivery. It is demonstrated that: (i) it is possible to separate and thoroughly characterize dendrons before conjugating them onto the nanoparticles and (ii) dendrons coated onto MeSiNP affect the mucoadhesive properties of the particles. This suggests that the system holds strong potential as a possible oral delivery carrier.

## Results and Discussion

### Novel Synthetic Route for Preparation of Dendrons

**Figure 1:**
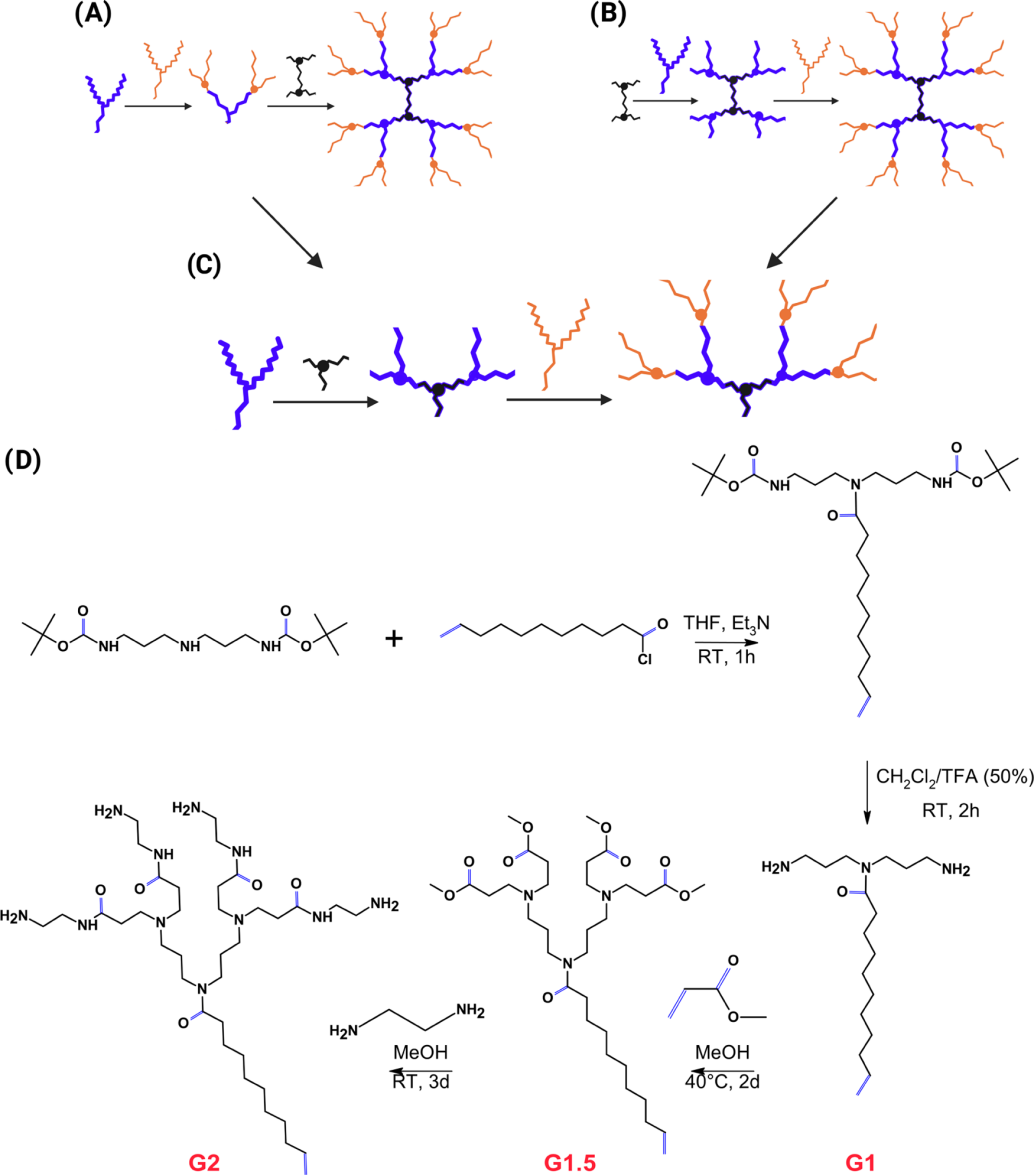
Dendrons’ synthetic route. (A) Convergent approach for the synthesis of dendrons, where shorter branches are joined to form larger dendrons; (B) divergent approach for the synthesis of dendrons, where the dendron is extended by a series of Michael addition and amidation reactions; (C) novel approach, used in this work, for the synthesis of dendrons, which combines both the divergent and convergent approaches. This is done by joining two shorter branches at first (polyamine head and the fatty acid) yielding a generation 1 (G1) dendron, which can be further extended by a series of Michael addition and amidation reactions; and (D) principal steps in the synthesis of generation 1 (G1) dendrons where the initially BOC-protected bis(3-aminopropyl) amine reacts with the acid chloride through a nucleophilic acyl substitution, followed by the acid chloride reacting with a secondary amine to give a tertiary amide. This is followed by a deprotection step in 50%TFA/DCM. Finally, the dendrons can be further increased by Michael addition (resulting in G1.5 dendrons) and amidation reactions (resulting in G2 dendrons).

Historically, dendrons and dendrimers have been synthesized in various sizes and forms. There are two different methods for the synthesis of dendrimers: the convergent and the divergent method. In the convergent approach, seen in Figure 1A, multiple dendrons are initially synthesized and are then reacted into a core to finally have a multibranched dendrimer.^20^ The second approach, the divergent method, involves the repetition of multiple addition–reduction steps for dendrimer growth from a core into a multibranched polymer, seen in Figure 1B.^26^ In our case, dendrons were synthesized by combining both of the methodologies, as shown in Figure 1C.

The synthesis starts with applying the convergent strategy where the dendron’s “head” (BOC-protected bis(3-aminopropyl) amine) reacts with the “tail” (acid chloride), as shown in Figure 1D. Once the BOC group has been removed, the synthesis continues by following the divergent approach, which is a repetition of the Michael addition and amidation reactions, where the dendrimer can be expanded in size and generation, as shown in Figure 1D.^26^ This sequence of reactions leads to amine-terminated full generation (e.g., G1 and G2) or carboxyl-terminated half-generation dendrons (G1.5). Therefore, thorough characterization through ^1^H NMR and MS is especially important when using the divergent nontrivial approach.^18^ Likewise, in the synthesis of dendrimers, several side reactions have been reported, such as partial surface functionalization or trailing generations (where some branches are shorter than others) as a result of the poor removal of methyl acrylate residues.^25^ Applying the commonly used method for conjugation of dendrons onto MeSiNP can also lead to several side reactions, resulting in a completely different coating than expected, such as the formation of cyclic amides, which would prevent further branching of dendrons.^25^ In this work, G1 dendrons were analyzed by ^1^H NMR and MS, shown respectively in Figures S1 and S2, displaying their successful synthesis.

After optimization and workup, the synthesis of G1 dendrons resulted in an 80% yield. In the literature, there are only a few reported dendron structures or synthesis methods. Synthesis of similar structures to our G1 dendrons, e.g., aliphatic polyamines, which are usually used as cross-linkers in the preparation of hydrogels or other supramolecular structures, are reported to give a yield of 70%.^27,28^ Contrarily, for the synthesis of structures used for the preparation of azido-PAMAM dendrons, with the main difference between the two being that the tail attached to the primary amine in our case was a 10-undecenoyl chloride instead of a *p*-xylene diazide, the obtained high yield is in the range of 87%.^29^ The major loss in our case was found to be in the workup after the deprotection process. This is likely due to the amphiphilic nature of the molecule, which causes self-assembly in aqueous solution into micelles, and could be visibly observed as an emulsion in the solution during the deprotection workup, but most of the solution could be recovered by treating the mixture with ethanol (yield: 80%).^30^ For future scaling up, the production of these G1 dendrons, determination of their critical micelle concentration will be required to evaluate the addition of other surfactants to reduce the loss of material and thereby further improve the yield.

### Dendron Interaction with Mucin Affects the Mucin Layer Flexibility

**Figure 2:**
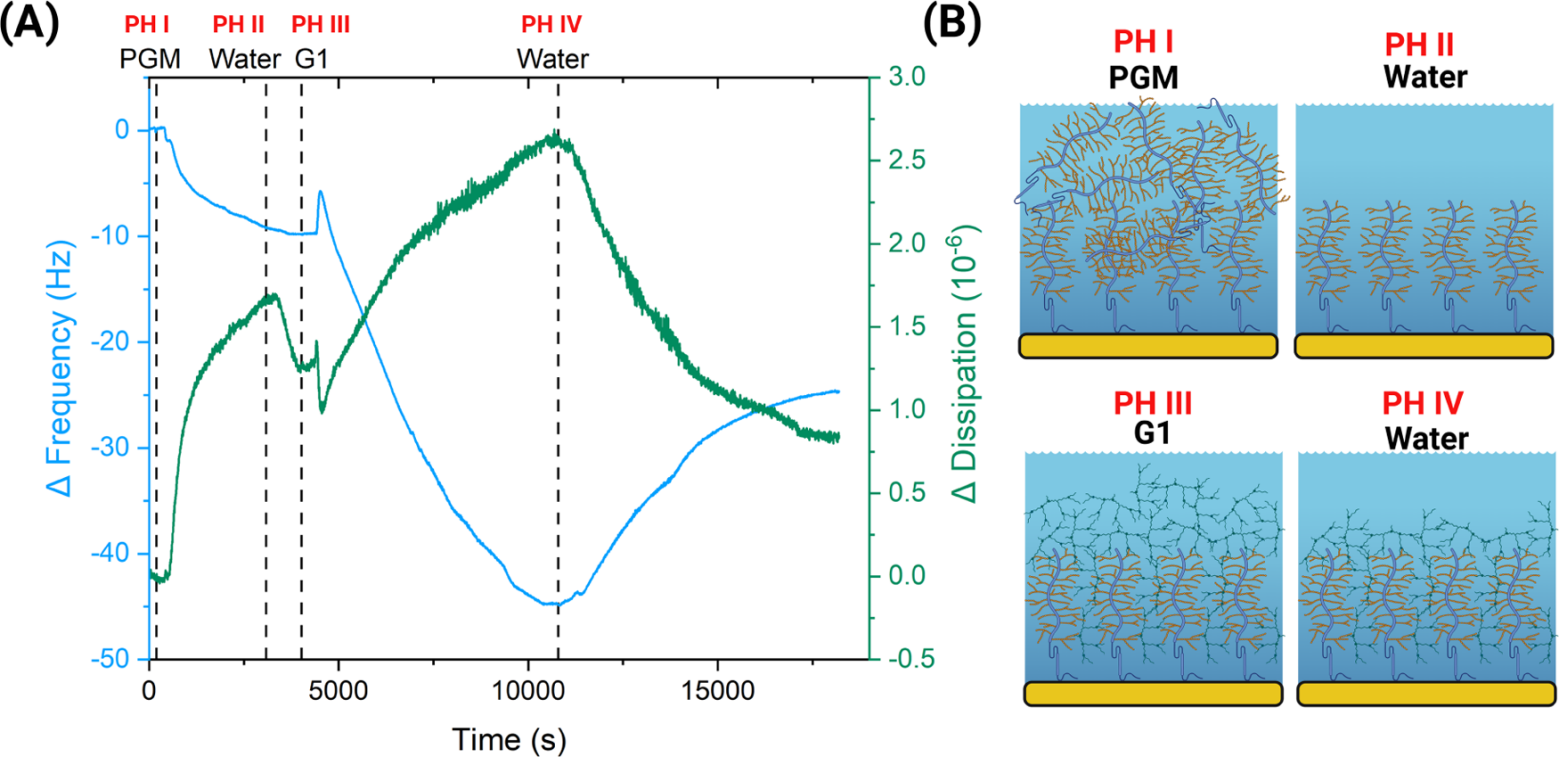
Dendron–mucin interaction. (A) Representative quartz crystal microbalance with dissipation (QCM-D) results display the interactions of dendrons with a layer of mucin conjugated to the gold surface as illustrated. The graph shows the interaction between the mucin layer and the generation 1 (G1) dendrons in pH 6.5 ultrapure water at 25 °C through the change in frequency (Δ*f*) in light-blue and dissipation (Δ*D*) in green for the fifth overtone as a function of time. Phase I: the formation of a physically adsorbed mucin layer. Phase II: rinsing with ultrapure water. Phase III: addition of G1 dendrons. Phase IV: rinsing with ultrapure water. (B) Illustration of the anticipated structure of the layer adsorbed to the surface shows the adsorbed layer of mucin onto the gold sensor (yellow) and its interaction with generation 1 (G1) dendrons (each step corresponds to a dashed line in (A)). (*N* = 3).

Various methods can be used to characterize and screen the interactions between excipients, such as dendrons with varying properties, and mucin prior to the selection of lead components and subsequent conjugation onto the nanoparticles. In Figure 2, we display the interactions between G1 dendrimers and porcine gastric mucin (PGM) using a quartz crystal microbalance with dissipation (QCM-D). QCM-D measures the frequency and dissipation changes, and while frequency changes depend on the mass on the sensor, dissipation changes can be used for the determination of the rigidity of the layer at the sensor surface.^31^

It is shown that a PGM layer can be formed on the sensor by physical adsorption (phase I). After washing and removal of loosely bound PGM (phase II), the exposure to a G1 solution (phase III) results in a change in both frequency (Δ*f*) and dissipation (Δ*D*). While a decrease was observed in the frequency, the dissipation increased, indicating a mass uptake of G1 and a change in the mucin layer stiffness. Specifically, the change in Δ*f* from −10 to −40 Hz after addition of the G1 solution suggests an increased layer thickness on the gold surface due to interactions between mucin and the dendrons.^32^ Further, it was evident that after the last washing step with ultrapure water (phase IV), a net difference of about 10 Hz was observed when compared to the value prior to addition of the G1 dendron. This suggests a strong interaction between PGM and G1, as the G1 could not be washed off.^32^ Furthermore, the change in Δ*D* through the experiment suggests that G1 dendrons interacted with the mucin network and affected its overall flexibility. The interaction was further confirmed by a control experiment, as shown in Figure S3C,D, where we studied the adsorption of G1 only on the sensor. The results show how, initially, there is an adsorption of the dendrons on the sensor shown by the change in frequency, but it is then quickly and completely washed off by ultrapure water with a net change of 0. This confirms that the changes in frequency and dissipation observed above are completely due to the interaction between mucin and dendrons and not the adsorption of mucin on the sensor. When compared to results reported in the literature, it remains clear that Δ*f* varies depending on the solvent.^33,34^ As explained by Wan et al.,^33^ this is probably due to the conformation that mucin adopts in the solvent, which may reduce or increase the physical adsorption on the gold-coated crystals based on the charge of the protein.^33^ Similar results were observed by Bravo-Osuna et al.^35^ when measuring the interaction between transmembrane ocular mucins and dendrimers. In their work, the PAMAM dendrimers were successfully benchmarked for mucoadhesion against chitosan, well known for its interaction with mucins.^35^ Our results on dendron–mucin interactions confirm what was previously observed with PAMAM dendrimers^35^ that they strongly interact due to the high density of active amine groups located on the dendron’s surface. QCM-D shows that there is a change in the stiffness of the mucin layer adsorbed to the surface. This is most likely due to: (i) the electrostatic interaction and hydrogen bonding between the mucin and the dendrons^35^ and (ii) the mucin network, which is a dynamic structure that can interact with molecules within it.^36^

**Figure 3:**
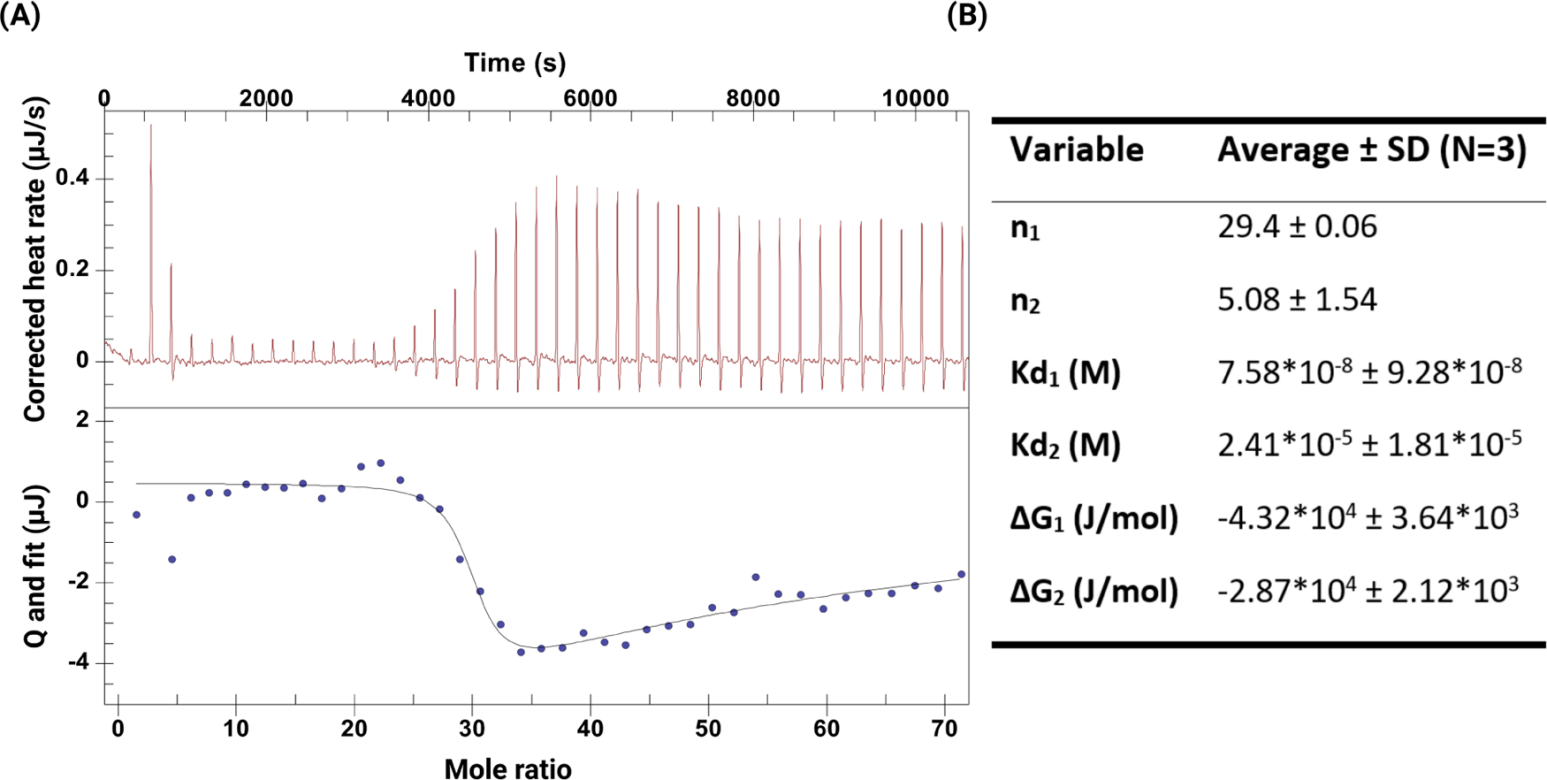
Dendron–mucin thermodynamic interaction. (A) Representative isothermal titration microcalorimetry (ITC) titration profile of raw heats of binding (A, top panel) for mucin (0.3 mg/mL) titrated with generation 1 (G1) dendrons (0.052 mM) in pH 6.5. Ultrapure water at 25 °C. (A) Lower panel shows the multiple binding sites’ fit of the binding data of integrated heats of binding, excluding dilution effects using NanoAnalyze software. (B) Average ± SD of various thermodynamic parameters extracted from the individual fitting curves (*N* = 3).

The mucin–dendron interaction was further quantified by isothermal titration calorimetry (ITC), as shown in Figure 3.

The individual titration graphs showed that the interactions between mucin and G1 dendrons were endothermic as the heat was released.^37^ The thermodynamic parameters were extracted from the best fit for each of the three individual titrations and are shown in Figure 3B. The heat traces were best modeled after a two-site binding model, which suggested that the dendrons might be interacting with PGM at two nonidentical and independent binding sites. The value reported in Figure 3B showed very different stoichiometry values between the two sites (*n*_1_ and *n*_2_), as the first had a stoichiometry of 29 (dendrons to mucin), while the second one had a stoichiometry value of 5 (dendrons to mucin), likely ascribed to the mucin structure.^38^ Mucin is a highly glycosylated protein, mostly with O-linked oligosaccharides between carbohydrates (mainly N-acetylgalactosamine, but also galactose and sialic acid) and serine or threonine, but also N-glycosidic linkages between the carbohydrates and asparagine.^39^ Although the used mucin material is partly purified, the distribution of mucins in the sample both in terms of glycosylation patterns and chain length represents the heterogeneous nature of the biological material. Therefore, the two-site binding model may be explained by the fact that the dendrons, given their small size, are able to interact differently with sugar moieties as opposed to interacting with the backbone. Also, different glycosylation patterns may result in different binding stoichiometries represented by *n*_1_ and *n*_2_.^38^ Importantly, a 1000-fold difference between the two obtained binding affinities was observed, the first one having a *K*_d_ value of 3 orders of magnitude larger than that for the other binding site, 7.56 × 10^–8^ and 2.41 × 10^–5^ M, respectively. Finally, the Gibbs free energy (Δ*G* = Δ*H* – *T*Δ*S*) was negative at *T* = 298 K (25 °C) for both binding sites, suggesting that the interaction between the G1 dendrons and mucin was thermodynamically favorable. There is a lack of studies between mucin and dendrons using ITC, yet a few studies evaluate the interaction between mucin and the cationic polymer chitosan. The results by Menchicchi et al.^40^ on the interaction between mucin and chitosan also showed multiple sites and thermodynamically favorable binding, similar to our findings (Figure 3A).

In conclusion, using different characterization techniques, we demonstrated a strong interaction between the G1 dendrons and mucin layer, most likely due to the electrostatic interactions and hydrogen bonding between the two. Employing different complementary techniques was proven valuable for the characterization of the interaction between new excipients and mucin.

### Characterization of Generation 1 Dendron-Decorated MeSiNP

**Figure 4:**
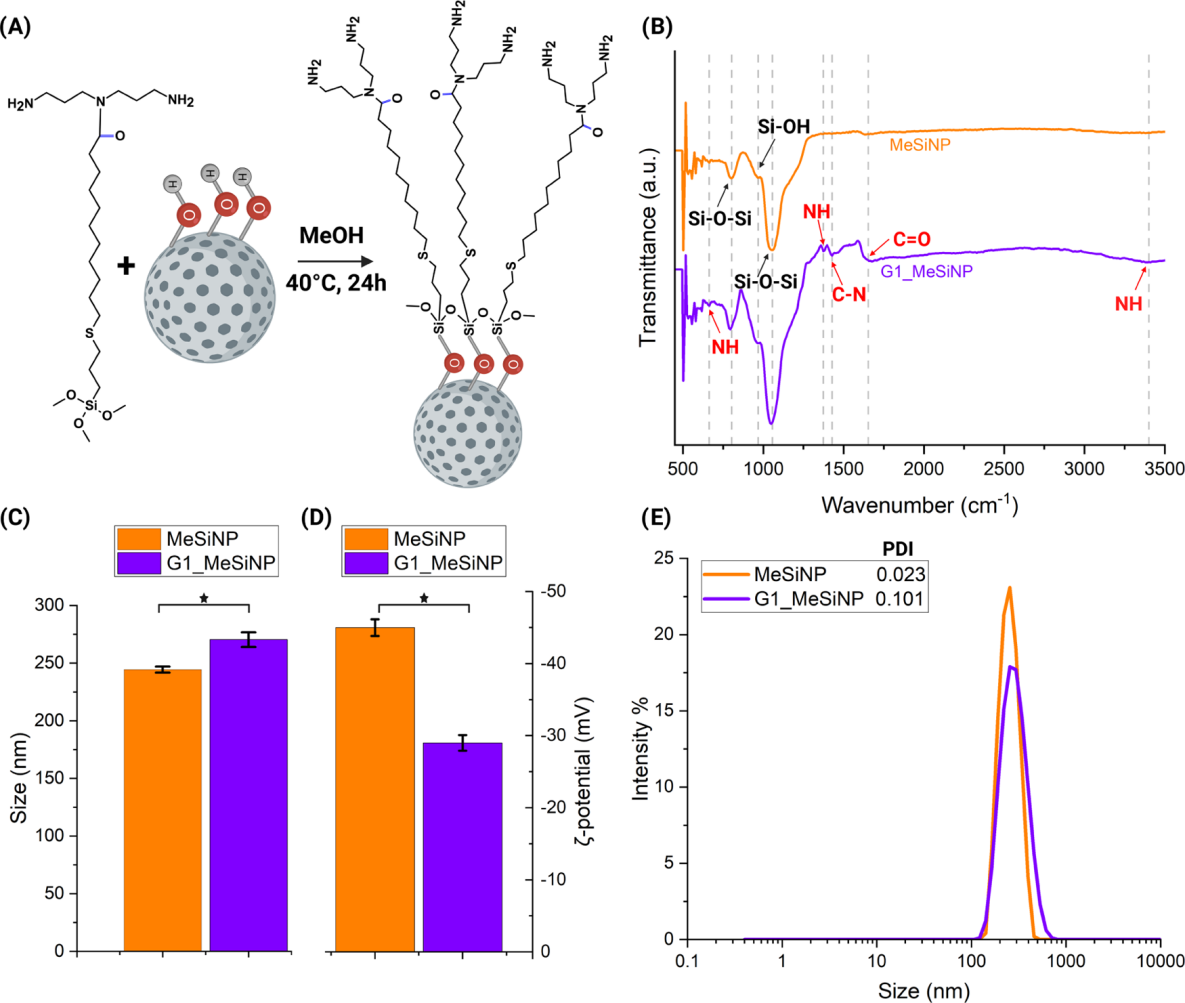
Conjugation and characterization of generation 1 dendrons on mesoporous silica nanoparticles. (A) Shows the conjugation of generation 1 (G1) dendrons to mesoporous silica nanoparticles (MeSiNP), where the hydroxyl groups on the nanoparticles, by silanization, displace the alkoxysilane group on the dendrons for formation of a covalent −Si–O–Si– bond. (B) The Fourier transform infrared spectroscopy (FTIR) superimposed the spectra of MeSiNP (orange) and G1_MeSiNP (purple) powders displayed as transmittance versus wavenumber (each dashed line corresponds to a specific functional group peak, as shown). (C) Hydrodynamic diameter of the nanoparticles for MeSiNP (orange) and G1_MeSiNP (purple). (D) ζ-potential for MeSiNP (orange) and G1_MeSiNP (purple). (E) Example of the distribution and intensity of the particles and their polydispersity index (PDI). Results are shown as the average ± SD (*N* = 3 *n* = 3), *p < 0.05, and the final concentration of nanoparticles was 0.1 mg/mL in ultrapure water.

After thorough characterization of the interactions between G1 dendrons and mucin, the G1 dendrons were conjugated onto the MeSiNP, through a silanization reaction, as shown in Figure 4A. The successful coating was determined using Fourier transform infrared spectroscopy (FTIR). From Figure 4B, the difference in peaks before and after the conjugation was clear. Similar to what was previously reported in the literature, the peaks at 803, 968, and 1054 cm^–1^ were representative of MeSiNP nanoparticles, while the ones observed at 664, 1374, 1429, 1680, and 3470 cm^–1^ represented functional groups introduced by the G1 dendrons, as reported on the graph, suggesting a successful coating.^22,41^ Successful conjugation was further confirmed by dynamic light scattering (DLS), measuring the hydrodynamic size and laser Doppler electrophoresis measuring the ζ-potential of MeSiNP and G1_MeSiNP (Figure 4C,D).

To demonstrate that the coating was complete, we conjugated the G1 dendrons on the MeSiNP at different (w/w) ratios, as shown in Table S1. The ζ-potential showed that complete coverage was considered obtained at a 1:5 ratio, as no further change in the ζ-potential was observed at larger ratios. The average diameters for MeSiNP and G1_MeSiNP were 244.4 ± 2.6 and 270.4 ± 6.4 nm, respectively, showing how the modification affected the hydrodynamic size of the nanoparticles (*N* = 3, *n* = 3). When comparing the ζ-potential, the data showed that the conjugated samples G1_MeSiNP (−29.0 ± 1.08 mV) was less negatively charged than the MeSiNP (−45 ± 1.15 mV). This is expected to be due to the −NH_2_ terminating group on the G1 dendrons adding a positive charge to the −OH on the MeSiNP.^22^ Lastly, the size distribution profile and the polydispersity index (PDI), as shown in Figure 4E, demonstrated a highly monodispersed sample, even after conjugation resulting in a PDI of 0.102.

**Figure 5:**
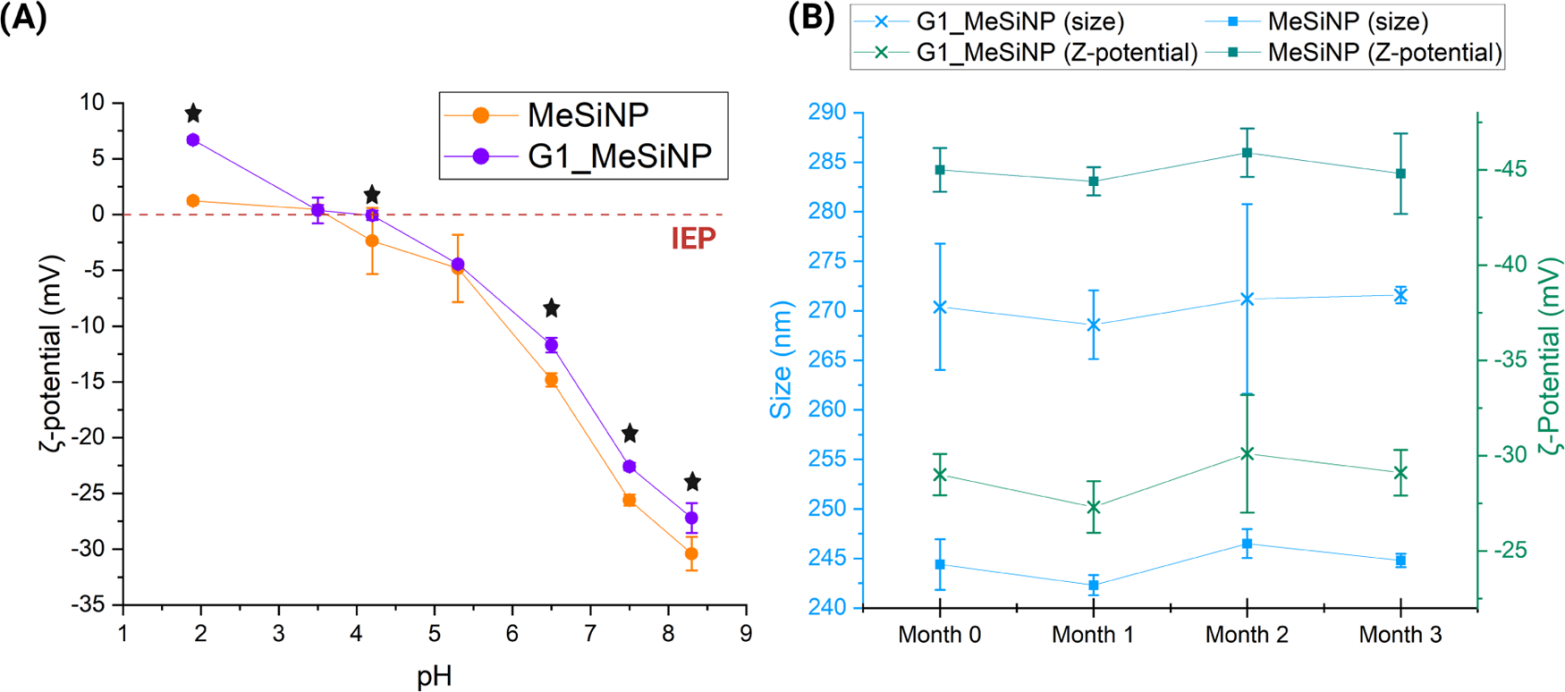
Particle stability. (A) Change in ζ-potential as a function of different pH values for mesoporous silica nanoparticles (MeSiNP) (orange) and generation 1 (G1) dendron-coated MeSiNP (purple) in phosphate-buffered saline (PBS) buffer (nanoparticles’ final concentration 0.1 mg/mL) and their isoelectric point (IEP) shown with the dashed line. (B) Size and ζ-potential of the MeSiNP and the G1_MeSiNP for 3 months after storing the particles at 8 °C in a 15 mL centrifuge tube (concentration 1 mg/mL) at pH 6.5. The graph shows the hydrodynamic diameter of the nanoparticles (blue) and the ζ-potential (green) of the MeSiNP (cross) and G1_MeSiNP (square). Results are shown as the average ± SD (*N* = 3, *n* = 3), *p < 0.05, and the nanoparticle final concentration was 0.1 mg/mL.

Furthermore, the chemical (conjugation) stability and the colloidal stability of the nanoparticles were evaluated by their change in ζ-potential and size over 3 months. The charge of MeSiNP and G1_MeSiNP is critical to study since nanoparticle behavior after administration and exposure to different pH values in the GIT would affect the overall charge of the nanoparticles differently.^42^ It is evident that the nanoparticles in the buffer of pH 2–8.5 display different ζ-potentials at different pH values, as shown in Figure 5A. Respectively, the measurements at pH 8.3 (*p*: 0.0001), pH 7.5 (*p*: 0.0001), pH 6.5 (*p*: 0.0001), pH 4.2 (*p*: 0.0001), and pH 1.9 (*p*: 0.0001), showed that the coating has an effect on the particle’s charge (measured using an unpaired *t*-test). For pH 5.3 (*p*: 0.16) and pH 3.5 (*p*: 0.48), the values are not statistically different; however, at the IEP when it crosses the 0 mV ζ-potential threshold shown in Figure 5A, there is a significant difference between G1_MeSiNP and MeSiNP.

Also, the IEP, when the net charge of the dispersion is zero,^43^ is between pH 4 and 5 for the G1_MeSiNP and pH 3 and 4 for the bare MeSiNP. A difference was expected due to the presence of amines on the surface of the G1_MeSiNP, when compared to the IEP of MeSiNP, which is also in agreement with the literature.^43^ The difference in the net charge evident at different pH values highlights how the coating of the nanoparticles with the dendrons changes their properties. This interesting finding can be used to predict that by coating the nanoparticles with higher generations of dendrons, the IEP can be shifted toward higher values. This would allow better control of the nanoparticle charge at different sites in the GIT and thus their degree of mucin interaction. Figure 5B shows the colloidal stability of the nanoparticles over 3 months, as this needs to be considered when designing a DDS.^44^ The graph confirmed colloidal stability over time, as measured by the particle size and ζ-potential, after several months of storage at 8 °C in a 15 mL centrifuge tube (concentration 1 mg/mL), following conjugation of the dendrons to the surface. The size was around 270 nm and the ζ-potential around −29 mV for the G1_MeSiNP, and further, the PDI was in the range of 0.13 ± 0.02 for the G1_MeSiNP and 0.06 ± 0.02 for the MeSiNP samples, indicating that the nanoparticles neither aggregated nor lost their functionalization over time (MeSiNP size *p* = 0.12, G1_MeSiNP size *p* = 0.20; MeSiNP ζ-potential *p* = 1, G1_MeSiNP ζ-potential *p* = 0.59). Overall, results from DLS and FTIR showed that the conjugation characterization was in agreement with what was previously observed in the studies of PAMAM dendrimers coated on MeSiNP.^19,45^ Conclusively, we have shown a successful conjugation of G1 dendrimers to MeSiNP and their stability over time, which allows for further characterization of the system for mucoadhesion as a potential future DDS in nanoformulations.

### Coating Affects MeSiNP Interaction with Mucin and Mucus

**Figure 6:**
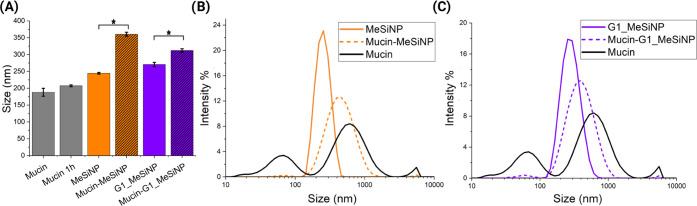
Nanoparticle interaction with mucin. (A) Mucin–particle interaction results from dynamic light scattering (DLS) measurements. The nanoparticle hydrodynamic diameter (mucin in gray, mesoporous silica nanoparticles (MeSiNP) in orange, and generation 1 (G1) coated MeSiNP in purple) before and after incubation with mucin mixed 50% v/v in ultrapure water. (B) Size distribution intensity profile of mucin (black), MeSiNP (orange), and MeSiNP–mucin (orange, dash). (C) Size distribution intensity profile of mucin (black), G1_MeSiNP (purple) and G1_MeSiNP–mucin (purple, dash). Results are shown as the average ± SD (*N* = 3 *n* = 3), *p < 0.05, and the nanoparticle final concentration was 0.1 mg/mL.

As described above, the dendrons interacted with mucin. However, when analyzing nonconjugated dendrons, it was not evident which groups interacted with mucin, the surface group amines, or the dendrons’ tail. Thus, the interaction of MeSiNP and G1_MeSiNP with PGM as well as mucoadhesion of the nanoparticles with porcine intestinal mucus (PIM) was determined. This information is important as it represents a more *in vivo-*like environment, as PIM is a complex viscous fluid, composed not only of mucin but also of inorganic salts, lipids, and other proteins, which interconnects to form a network with pores of various sizes.^46^ The mucoadhesion was first predicted through changes in the hydrodynamic size due to interactions between the nanoparticles and mucins. The data in Figure 6A analyzed for statistically significant differences (*p* < 0.05), showed an increase in the MeSiNP particle size from 244.4 ± 2.6 to 360.3 ± 5.9 nm after 1 h of incubation at room temperature (RT) with PGM, while an increase from 270.4 ± 6.4 to 312.1 ± 8.2 nm was evident for the G1_MeSiNP, both indicating interactions between mucin and the particles. Control samples in ultrapure water did not change the size, being 188.1 ± 11.7 nm at time 0 and 207.3 ± 2.6 nm after 1 h (*p* > 0.05).

These results are further confirmed by the intensity distribution showing a larger change in intensity for MeSiNP compared to that of G1_MeSINP before and after the incubation and a higher PDI value (MeSiNP 0.318, G1_MeSiNP 0.296), shown in Figure 6B,C. This differs from what is reported in the literature, where G1 dendrons are shown to increase mucoadhesion.^19^ In a study from Wang et al.,^19^ comparing how mucoadhesion changes with increasing dendrimer generation, the report did not compare the results with hydroxyl-terminated MeSiNP, as they synthesized them directly with an −NH_2_ surface group. Therefore, the two results do not contradict but rather complement each other. The strong mucoadhesion of dendrimers coated onto MeSiNP reported in the literature can be explained by the opposite charge attraction between mucin and dendrimer-coated nanoparticles.^15,19^ Having said that, mucoadhesion occurs not only due to opposite charge interactions but also via hydrogen bonding as well as steric interactions.^19,47^ This likely explains why we see a stronger mucin interaction with the bare nanoparticles compared to G1 dendron-coated particles, as the ζ-potential of the dendron-coated nanoparticles remains negative after conjugation. This suggests that the charge interaction does not have a strong effect on mucoadhesion in the case of G1 dendron-coated nanoparticles compared to noncoated particles.^19^ Therefore, we conclude that the charges of G1_MeSiNP and MeSiNP, even though significantly different and both negative, are equivalent in terms of interacting with the mucin. The main difference between G1_MeSiNP and MeSiNP is their surface functional groups. The alkoxy and hydroxyl groups on the MeSiNP create stronger hydrogen bonds compared to the hydrogen bonds formed by the amine on G1_MeSiNP.^48^ These two considerations can collectively explain the stronger interaction between MeSiNP and mucin.

**Figure 7:**
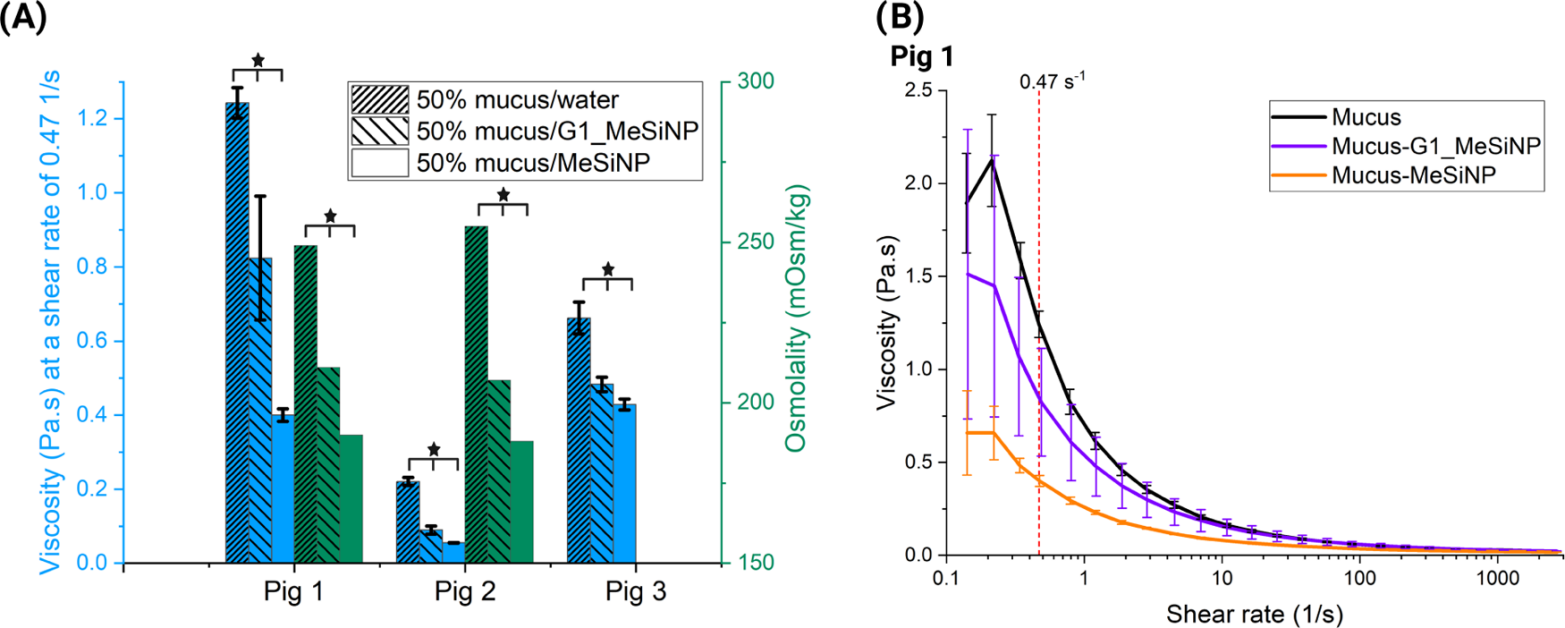
Nanoparticle interaction with mucus. (A) Rheology and osmolality measurements of different biological mucus samples incubated with mesoporous silica nanoparticles (MeSiNP) and generation 1 (G1)-coated MeSiNP. Viscosity (blue) obtained at a shear rate of 0.47 s^–1^ showed decreasing values upon addition of particles to the mucus samples: Mucus, mucus–MeSiNP, and mucus-G1_MeSiNP. Osmolality results (green) in two biological samples of mucus, mucus–MeSiNP, and mucus-G1_MeSiNP. All samples were 50% v/v in ultrapure water and the added particle concentration was 0.5 mg/mL. Results are shown as the average ± SEM, *p < 0.05, (*N* = 3, *n* = 3). (B) Shows biological samples for pig 1 of mucus (black), mucus–MeSiNP (orange), and mucus-G1_MeSiNP′(purple). Results are shown as the average ± SD, (*n* = 3).

In the rheological measurements performed to study the interaction of the nanoparticles with PIM, a similar behavior was observed by which the coating influenced the mucoadhesion. Incubation of PIM with the nanoparticles (50% v/v, nanoparticles in ultrapure water at a concentration of 0.5 mg/mL) decreased the viscosity when compared to the control (50% v/v in ultrapure water). Even though PIM, due to its biological origin and complexity, had different viscosities and resulted in high variation between samples, we were able to show consistent behavior across three different pigs. This was clear from averaging and comparing the results at a shear rate of 0.47 s^–1^ as deemed relevant for human applications,^49^ and shown in Figure 7A,B.

As also observed by the DLS measurements using mucin, coating impacts the interaction between the nanoparticles and PIM as the viscosity is considered significantly different (*p* < 0.05). The sample from pig 1 decreased in viscosity from 1.24 ± 0.04 to 0.82 ± 0.16 Pa·s for G1_MeSiNP and 0.40 ± 0.01 Pa·s for MeSiNP, in Figure 7B. A similar behavior was observed with the other two pigs, with significant (*p* < 0.05) differences between the different samples (Figure S5A pig 2: control 0.22 ± 0.01 Pa·s, G1_MeSiNP 0.09 ± 0.01 Pa·s, MeSiNP 0.05 ± 0.001 Pa·s; Figure S5B pig 3: control 0.66 ± 0.04 Pa·s, G1_MeSiNP 0.48 ± 0.01 Pa·s, MeSiNP 0.42 ± 0.01 Pa·s). The decrease in viscosity was most likely due to interactions of the nanoparticles with mucin, which agreed with the DLS data. Moreover, it could be speculated that the MeSiNP might also mechanically disrupt the mucin network upon shear, hence decreasing the bulk viscosity of the mixture. Mucin (black curve) presents the classic viscoelastic profile for a polymer chain, while the addition of the MeSiNP and G1_MeSiNP resulted in a decrease in viscosity, which is shown as a shear thinning-like fluid plot.^50^ The interaction between the nanoparticles and mucus, as described before, is probably mostly due to hydrogen bonding. We hypothesize that in this case, the interaction between mucus and nanoparticles can be considered disruptive, meaning that the nanoparticles interact with and break apart the mucin polymeric network by disrupting the noncovalent mucin–mucin interactions. Thus, decreasing the overall viscosity of the mucin network. The decrease in viscosity, which is hypothesized to be due to interference of the particles with the mucin network, could also be the result of the mechanical displacement of the mucins, related to the large size of the nanoparticles (size >250 nm). The results additionally showed how a decrease in osmolality corresponds to a decrease in viscosity (pig 1: control 0.22 ± 0.01 Pa·s and 249 mOsm, G1_MeSiNP 0.09 ± 0.01 Pa·s and 211 mOsm, MeSiNP 0.05 ± 0.001 Pa·s and 190 mOsm; pig 2: control 0.66 ± 0.04 Pa·s and 255 mOsm, G1_MeSiNP 0.48 ± 0.01 Pa·s and 207 mOsm, MeSiNP 0.42 ± 0.01 Pa·s and 188 mOsm), as previously reported by Seeliger et al.^51^ The change in osmolality could be due to the interaction and adsorption (either on the surface or in the pores) between the nanoparticles and the inorganic salts and ions present in the PIM.^52^ This would also explain why there is a bigger change observed with the MeSiNP compared to that with the G1_MeSiNP, as they have a more available surface area for interactions and similarly ions have easier access to the pores. Overall, we can conclude that the dendrimer coating affects the interaction of the nanoparticles with mucin and PIM.

## Conclusions

This work shows the potential to surface-coat MeSiNPs with dendrons for use as a new oral mucoadhesive delivery system for oral administration.

Importantly, a novel route for the synthesis of G1 dendrons was introduced by combining the two most well-known dendron synthesis pathways (convergent and divergent). We showed that it is possible to separate the synthesis of dendrons from that of the nanoparticles, avoiding any possible complications due to trailing dendron generations when coating directly on the nanoparticles. Splitting the synthesis of dendrons and particles additionally allows for the coating of multiple types (e.g., G1 PAMAM and G1 PAMOL dendrons) and maybe even multiple generations of dendrons on the same nanoparticle for tailoring the functionality of the drug delivery system.

Further, the particles′ molecular interactions with mucin were proven and evaluated through structural changes and thermodynamic binding parameters, overall showing that G1 dendrons represent interesting candidates as oral delivery excipients. Data suggest that dendrons interacted with and changed the stiffness of a mucin layer, and the interaction was shown to occur through binding to multiple sites. Although nontrivial, we proved that conjugation of G1 onto MeSiNP was successful as indicated by changes in the size, charge, and the isoelectric point of the DDS, after the conjugation of dendrons to the particles. The interaction was further confirmed in studies using mucin as well as intestinal mucus isolated from pigs, as size measurements and rheology showed that both the bare and coated nanoparticles interacted with the mucin/mucus but that the coating dictates the affinity. Further, it was clear that the bare nanoparticles decreased the viscosity and osmolality more when compared to the effects of the coated nanoparticles, additionally confirming that the coating influences particle–mucus interactions. Thus, this study showed that it is possible to study the mucoadhesion of excipients before conjugation to nanoparticles, which is extremely useful in the case of dendrons, dendrimers, or similar polymers, as they could be functionalized and tested with various terminal groups before selecting the best candidate for the conjugation.

Observed in their totality, these results show the feasibility of a new synthesis approach for preparing dendron-coated nanoparticles as well as the potential to prepare a mucoadhesive oral delivery system by coating mucoadhesive dendrons onto MeSiNP supplemented by the fact that preconjugation studies of the coating excipients translates to findings after conjugation. This paves the way for further tailoring of dendrons for improved specificity and use in drug delivery.

## Methods

### Materials

Bis(3-aminopropyl)amine, undecylenic acid, (3-mercaptopropyl)trimethoxy silane, mesoporous silica nanoparticles (MeSiNP), methyl acrylate, ethylenediamine, mucin from porcine stomach Type III (PGM), phosphate-buffered saline (PBS, 0.01 M phosphate buffer, 0.0027 M potassium chloride and 0.137 M sodium chloride, pH 7.4, at 25 °C), dimethyl sulfoxide (DMSO), dichloromethane (CH_2_Cl_2_), dimethylformamide (DMF), tetrahydrofuran (THF), triethylamine (Et_3_N), and trifluoroacetic acid (TFA) were purchased from Sigma-Aldrich (St. Louis, MO) and used as received unless otherwise stated. Solvents were HPLC grade and used as received. Thin-layer chromatography was carried out using silica plates on aluminum (Silica 60-F254, 6 0.2 mm layer thickness, Merck, Darmstadt, Germany) with detection under UV light and, if required treatment with 1% (w/v) solution of ninhydrin in ethanol. ^1^H NMR spectra were obtained on a 500 MHz NMR (Bruker, Billerica, MA) apparatus. Chemical shifts are reported in parts per million (ppm) downfield of tetramethylsilane (TMS) using the resonance of the deuterated solvent as the internal standard. The ESI mass spectra for the dendrons were recorded with a Q-Tof Ultima GLOBAL mass spectrometer (Micromass, Manchester, U.K.) equipped with a Z-spray source (Micromass, Manchester, U.K.). Ultrapure water was used throughout the experiments and obtained from PURELAB flex (ELGA, High Wycombe, U.K.).

### Synthesis and Characterization of Dendrons

#### Polyamine Protection (1)

Dendrimer protection was performed as previously shown by Pittelkow et al.^27^ Briefly, bis(3-aminopropyl)amine (0.05 mol) and *t*-butyl phenyl carbonate (1.1 equiv per primary amino group) were added to 50 mL of DMSO and stirred for 24 h at room temperature (RT). The solution was poured into 1 L phosphate buffer (0.025 M K_2_HPO_4_ and 0.025 M NaH_3_PO_4_). The pH was adjusted to 3 with aqueous H_2_SO_4_ (2 M) and the mixture was extracted twice with CH_2_Cl_2_ (250 mL). The aqueous phase was isolated and made alkaline (pH > 10) with aqueous NaOH and extracted three times with CH_2_Cl_2_. The organic phase was isolated and dried with Na_2_SO_4_, filtered, and concentrated by applying vacuum on a rotary evaporator. Yield: 16.5 g (99.5%).

#### 10-Undecenoyl Chloride Synthesis (2)

Undecylenic acid (0.05 mol) was converted into the acid chloride by reaction with oxalyl chloride (1.1 equiv) with a catalytic amount of DMF (5 mL) in THF (100 mL). The reaction mixture was stirred for 2 h at RT. The solution was concentrated by applying vacuum on a rotary evaporator. Yield: 10 g (99%), ^1^H NMR (CDCl_3_) in Figure S1.

#### Synthesis of *tert*-Butyloxycarbonyl (BOC)-Protected Generation 1 PAMAM Dendron (3)

The protected polyamine (1) was reacted with the acid chloride (2) (0.9 mol equiv) in THF (100 mL) as the solvent and Et_3_N (3 mol equiv of polyamine) as the base. The solution was filtered and concentrated by applying vacuum on a rotary evaporator. Yield: 10.6 g (98.6%).

#### Generation 1 PAMAM Dendron Deprotection (4)

Deprotection of the BOC-protected amine (3) was done with CH_2_Cl_2_ and TFA (50/50%) and stirred at RT for 2 h. The reaction was monitored via the Kaiser test (1% (w/v) ninhydrin in ethanol). Upon completed conversion of the N-BOC-protected amine, deionized water (40 mL) and NaOH (12 M) were added to the flask slowly until the pH reached 10 or above, monitored using phenolphthalein used as an indicator in the titrations. The crude material was subsequently extracted by adding ethanol to break micelle formation. Lastly, the organic layer was dried with Na_2_SO_4_, filtered, and concentrated under vacuum on a rotary evaporator. The mixture was dissolved in CH_2_Cl_2_/MeOH/Et_3_N (85:10:5) and purified on a silica gel (63–200 μm) by flash column chromatography monitored on TLC plates by the Kaiser test (1% (w/v) ninhydrin in ethanol). Yield:4.9 (77.3%). ^1^H NMR (CDCl_3_) in Figure S1 and MS in Figure S2.

#### Thiol Functionalization of PAMAM Dendrons (5)

(3-Mercaptopropyl)trimethoxy silane (1 mol equiv) is added to G1 PAMAM (4) solution dissolved in chloroform (10% w/v), which was flushed with nitrogen for 10 min prior to the addition. The reaction mixture was illuminated with a halogen light source (100 W) under stirring for 48 h at RT under a N_2_ atmosphere. The solvent was removed in vacuum on a rotary evaporator. Yield: 6.85 g (84.3%).

### Mucin–Dendron Interaction Characterization

For mucin–dendron interaction measurements, the samples were dissolved in ultrapure water, sonicated, and degassed before measurements with a Bandelin Sonorex (BANDELIN electronic GmbH & Co. KG, Berlin, Germany). Measurements were carried out in ultrapure water at pH 6.5, as it is reported that all primary amino groups of PAMAM dendrons are protonated at this pH, promoting electrostatic interactions with the negatively charged sialic acid on PGM.^53^

#### QCM-D

Quartz crystal microbalance with dissipation (QCM-D) measurements was performed as previously shown by Wan et al.^33^ with an E4 system from Q-Sense (Gothenburg, Sweden) using gold-coated quartz crystals with a fundamental frequency of 4.95 Hz (QSX301, Q-Sense, Gothenburg, Sweden). Sensors were rinsed using 2% (w/v) sodium dodecyl sulfate and then washed with ultrapure water and ethanol, followed by drying using N_2_ gas. Experiments were conducted at 25 °C using a flow rate of 50 μL/min in a flow mode. Both changes in frequency (Δ*f*) and energy dissipation factor (Δ*D*) at the 3rd, 5th, 7th, 9th, 11th, and 13th overtones were simultaneously recorded. Prior to depositing the mucin layer, ultrapure water was pumped through the flow cells to stabilize the *f* and *D* signals. Then, the mucin solution (0.3 mg/mL) in water was introduced into the QCM-D cells, allowing the deposition of a mucin layer onto the surface of the sensor. Once the *f* and *D* signals were stable, the flow was again changed to ultrapure water to remove any loosely bound mucin. After washing, dendrons dissolved in ultrapure water (0.26 mM) were introduced into the QCM-D cells to investigate the mucin–dendron interaction. Lastly, the flow was switched back to ultrapure water to remove any loosely bound dendrons until the *f* and *D* signals were stable. Similarly, to study the adsorption of dendrons onto the surface of the sensor: briefly, prior to depositing the dendron’s layer, ultrapure water was pumped through the flow cells to stabilize the *f* and *D* signals. Then, a solution of dendrons dissolved in ultrapure water (0.26 mM) was introduced into the QCM-D cells, allowing the deposition onto the surface of the sensor. Once the *f* and *D* signals were stable, the flow was again changed to ultrapure water to remove any loosely bound dendrons. A representative QCM-D graph is presented, and additional ones are in Figure S3 (*N* = 3). The data presented in the text are for the fifth harmonic overtone.

#### ITC

Isothermal titration calorimetry (ITC) was performed using a low-volume Nano ITC (TA Instruments, New Castle, DE) instrument with an active cell volume of 190 μL. The experiments were performed by injecting dendrons (0.052 mM) into mucin (0.3 mg/mL). Dendrons were titrated into mucin using an injection volume of 1.2 μL. The content of the reaction cell was stirred continuously at 300 rpm. The time interval between the injections was set to 250 s, allowing a complete equilibration of the system between the injections. Reference experiments, where the dendrons were injected into ultrapure water or where ultrapure water was injected into mucin produced constant, nonzero heat. Titrations were done at 25 °C. A representative graph is shown in the manuscript and additional replicates are in Figure S4. Thermodynamic parameters are presented as the average ± SD (*N* = 3).

### Dendron-MeSiNP Conjugation and Characterization

#### MeSiNP Conjugation

MeSiNPs (200 nm, pore size 4 nm) were dispersed in MeOH (10 mL) using ultrasonication (ultrasonic bath, BANDELIN electronic GmbH & Co. KG, Berlin, Germany) for 15 min. Dendrons (5 equiv of MeSiNP) were dispersed in MeOH (5 mL) using the ultrasonic bath for 15 min. The solutions were mixed and stirred for 24 h at RT. The beads were centrifuged for 5 min at 5000 rpm and washed three times each with MeOH and ultrapure water. The nanoparticles were then resuspended in ultrapure water, frozen in dry ice, placed under vacuum, and freeze-dried overnight.

#### Size and Surface Charge

The hydrodynamic radius, polydispersity index, and ζ-potential measurements of nanoparticles were determined at RT using a Malvern Zetasizer Nano ZSP (Malvern Instruments, Worcestershire, U.K.) in disposable microcuvettes (Malvern Instruments, Worcestershire, U.K.). Dynamic light scattering was done using a volume of 100 μL and a 173° angle of detection with 11 runs of 10 s/run and three measurements. The ζ-potential was investigated by laser Doppler electrophoresis using the Zetasizer Nano ZS (Malvern Panalytical). A volume of 700 μL of the sample was measured in folded capillary cells (Malvern Panalytical) with 10 runs in 3 measurements. The conjugated nanoparticles were redispersed in ultrapure water to a concentration of 0.1 mg/mL and sonicated for 15 min prior to measurement. For stability measurements, the samples were stored at 8 °C in a 15 mL centrifuge tube (concentration 1 mg/mL) wrapped in aluminum foil to protect them from light for up to 3 months.

#### Isoelectric Point

The titration measurements for isoelectric point studies were performed by measuring the ζ-potential as a function of pH using a Malvern ZetaSizer Nano-ZS. The samples were dispersed in PBS and the pH was adjusted (8.3, 6.5, 5.3, 4.2, 3.5, and 1.9) by titrating with HCl (0.01 M) or NaOH (0.01 M). The samples were dispersed by sonication for 15 min prior to measurements at RT.

#### Conjugation Efficiency

Fourier transform infrared (FTIR) spectroscopy was done on dry samples (MeSiNPs and dendron-conjugated MeSiNPs) and the spectra were recorded with an FTIR (Perkin-Elmer, Waltham, MA) in the range between 400 and 4000 cm^–1^ at RT.

### Mucoadhesion Studies

#### Mucus Isolation^54^

Intestines from healthy fasted (18–24 h) gilts (40–60 kg, 3–4 months, Danish Landrace) were obtained after experimental surgery. Immediately after euthanization, up to 5 m jejunum was isolated distal to the ligament of Treitz. Sections were opened by a latitude cut and porcine intestinal mucus (PIM) was isolated by gently scraping the mucosal surface. Mucus was kept on ice at all times and stored at −20 °C until use. Procedures were according to the authorization by Danish Veterinary and Food Administration (license number DK-13-oth-931833).

#### Mucin–Particle Interaction^19^

The hydrodynamic radius of nanoparticles was measured at RT using a Malvern Zetasizer. MeSiNP and G1_MeSiNP were tested. A mucin solution was prepared (1%, w/v) in ultrapure water, and the dispersion was prepared by mixing and sonication and centrifuged until the particle size was less than 240 nm as detected by DLS measurements. For size determination, equal volumes of the mucin dispersion and the nanoparticles were incubated for 1 h (final nanoparticle concentration of 0.1 mg/mL).

#### Rheological Properties

An ARES-G2 rheometer equipped with a Peltier plate and truncated cone (1°, 40 mm from TA Instruments, New Castle, DE) was used for the rheological measurements. The samples were analyzed using a continuous ramp step with increasing shear rates from 0.1 to 3000 s^–1^ sampling 5 points per decade at 25 °C. Prior to measurements, a conditioning step of 5 min at 50 s^–1^ was applied. Samples were mixed in ratios of 1:1 (v/v) mucus/water, mucus/MeSiNP, and mucus/G1_MeSiNP (final particle concentration was 0.5 mg/mL). Samples were prepared on the day of analysis and stored for 1 h before measurements at RT. Measurements were performed on PIM obtained from three different pigs in 3 replicates for each condition (*N* = 3, *n* = 3). Data are reported as the average ± SEM at a shear rate of 0.47 s^–1^, reported as relevant to the conditions in the human intestine.^49^

#### Osmolality

The osmolality of mucus/water, mucus/MeSiNPs, and mucus/G1_MeSiNPs were measured by freezing point depression using a Camlab Roebling microosmometer (Cambridge, U.K.) after 1 h incubation at RT.

### Data and Statistical Analysis

Data analysis was performed with OriginPro (Version 2021b, OriginLab Corporation, Northampton, MA). Data are presented as average and standard derivation (SD) where *n* represents the number of repetitions within each sample and *N* represents the number of samples. Data from the rheology studies are presented as the average and standard error of the mean (SEM), where *n* represents the number of repetitions within each sample and *N* represents the number of samples. Statistical analysis was performed for statistically significant differences (*p < 0.05) with OriginPro using a *t*-test (for two independent populations) or one-way analysis of variance (ANOVA) (three or more independent populations) with Tukey’s and Bonferroni test. Figures were created with Biorender.com

## Supporting Information Available

NMR spectra; mass spectra of G1 and G2 dendrons; QCM-D measurements; ITC titration measurements; conjugation and characterization of generation 1 dendrons on mesoporous silica nanoparticles; and rheological measurements of nanoparticles interacting with mucus (PDF)

## Abbreviations

BOC: *tert*-butyloxycarbonyl
CDCl_3_: deuterated chloroform
CH_2_Cl_2_: dichloromethane
Δ*D*: dissipation
DCM: dichloromethane
DDS: drug delivery system
DLS: dynamic light scattering
DMF: dimethylformamide
DMSO: dimethyl sulfoxide
Et_3_N: triethylamine
Δ*f*: frequency
FTIR: Fourier transform infrared spectroscopy
Δ*G*: Gibbs free energy
G0: generation 0
G1: generation 1
G2: generation 2
G4: generation 4
GIT: gastrointestinal tract
Δ*H*: enthalpy
^1^H NMR: proton nuclear magnetic resonance
IEP: isoelectric point
ITC: isothermal titration microcalorimetry
MeSiNP: mesoporous silica nanoparticles
MS: mass spectrometry
PAMAM: polyamidoamine
PDI: polydispersity index
PGM: porcine gastric mucin
PIM: porcine intestinal mucus
QCM-D: quartz crystal microbalance with dissipation
Δ*S*: entropy
SD: standard deviation
*T*: temperature
TFA: trifluoroacetic acid
THF: tetrahydrofuran

## References

[ref1] Homayun B.; Lin X.; Choi H.-J. Challenges and Recent Progress in Oral Drug Delivery Systems for Biopharmaceuticals. Pharmaceutics 2019, 11, 129 10.3390/pharmaceutics11030129. 30893852 PMC6471246

[ref2] Zhou X.; Wu H.; Long R.; Wang S.; Huang H.; Xia Y.; Wang P.; Lei Y.; Cai Y.; Cai D.; Liu Y. Oral Delivery of Insulin with Intelligent Glucose-Responsive Switch for Blood Glucose Regulation. J. Nanobiotechnol. 2020, 18, 96 10.1186/s12951-020-00652-z. PMC7362448 32664978

[ref3] Wong C. Y.; Martinez J.; Dass C. R. Oral Delivery of Insulin for Treatment of Diabetes: Status Quo, Challenges and Opportunities. J. Pharm. Pharmacol. 2016, 68, 1093–1108. 10.1111/jphp.12607. 27364922

[ref4] Fonte P.; Araújo F.; Reis S.; Sarmento B. Oral Insulin Delivery: How Far Are We?. J. Diabetes Sci. Technol. 2013, 7, 520–531. 10.1177/193229681300700228. 23567010 PMC3737653

[ref5] Catic T.; Gojak R.; Djekic D. Disposal of Used Pens and Needles from Diabetes Patients Perspective. Mater. Socio-Med. 2020, 32, 267–270. 10.5455/msm.2020.32.267-270. PMC7879436 33628128

[ref6] Owens D. R. New Horizons — Alternative Routes for Insulin Therapy. Nat. Rev. Drug Discovery 2002, 1, 529–540. 10.1038/nrd836. 12120259

[ref7] Patel G.; Misra A. Oral Delivery of Proteins and Peptides: Concepts and Applications. In Challenges in Delivery of Therapeutic Genomics and Proteomics; Misra A., Ed.; Elsevier: London, 2011; Chapter 10, pp 481–529.

[ref8] Carino G. P.; Mathiowitz E. Oral Insulin Delivery1Abbreviations: GI, Gastrointestinal; IDDM, Insulin-Dependent Diabetes Mellitus; IU, International Units; NIDDM, Non-Insulin-Dependent Diabetes Mellitus; PIN, Phase Inversion Nanoencapsulation; ZOT, Zona Occludens Toxin. Adv. Drug Delivery Rev. 1999, 35, 249–257. 10.1016/S0169-409X(98)00075-1.

[ref9] Banerjee A.; Qi J.; Gogoi R.; Wong J.; Mitragotri S. Role of Nanoparticle Size, Shape and Surface Chemistry in Oral Drug Delivery. J. Controlled Release 2016, 238, 176–185. 10.1016/j.jconrel.2016.07.051. PMC5289391 27480450

[ref10] Cheng H.; Cui Z.; Guo S.; Zhang X.; Huo Y.; Mao S. Mucoadhesive versus Mucopenetrating Nanoparticles for Oral Delivery of Insulin. Acta Biomater. 2021, 135, 506–519. 10.1016/j.actbio.2021.08.046. 34487859

[ref11] Tieu T.; Alba M.; Elnathan R.; Cifuentes-Rius A.; Voelcker N. H. Advances in Porous Silicon–Based Nanomaterials for Diagnostic and Therapeutic Applications. Adv. Ther. 2019, 2, 1800095 10.1002/adtp.201800095.

[ref12] Shrestha N.; Shahbazi M.-A.; Araújo F.; Mäkilä E.; Raula J.; Kauppinen E. I.; Salonen J.; Sarmento B.; Hirvonen J.; Santos H. A. Multistage PH-Responsive Mucoadhesive Nanocarriers Prepared by Aerosol Flow Reactor Technology: A Controlled Dual Protein-Drug Delivery System. Biomaterials 2015, 68, 9–20. 10.1016/j.biomaterials.2015.07.045. 26253804

[ref13] Stead S. O.; Kireta S.; McInnes S. J. P.; Kette F. D.; Sivanathan K. N.; Kim J.; Cueto-Diaz E. J.; Cunin F.; Durand J.-O.; Drogemuller C. J.; Carroll R. P.; Voelcker N. H.; Coates P. T. Murine and Non-Human Primate Dendritic Cell Targeting Nanoparticles for in Vivo Generation of Regulatory T-Cells. ACS Nano 2018, 12, 6637–6647. 10.1021/acsnano.8b01625. 29979572

[ref14] Elnathan R.; Delalat B.; Brodoceanu D.; Alhmoud H.; Harding F. J.; Buehler K.; Nelson A.; Isa L.; Kraus T.; Voelcker N. H. Maximizing Transfection Efficiency of Vertically Aligned Silicon Nanowire Arrays. Adv. Funct. Mater. 2015, 25, 7215–7225. 10.1002/adfm.201503465.

[ref15] Florek J.; Caillard R.; Kleitz F. Evaluation of Mesoporous Silica Nanoparticles for Oral Drug Delivery – Current Status and Perspective of MSNs Drug Carriers. Nanoscale 2017, 9, 15252–15277. 10.1039/C7NR05762H. 28984885

[ref16] Desai D.; Prabhakar N.; Mamaeva V.; Karaman D. Ş.; Lähdeniemi I. A.; Sahlgren C.; Rosenholm J. M.; Toivola D. M. Targeted Modulation of Cell Differentiation in Distinct Regions of the Gastrointestinal Tract via Oral Administration of Differently PEG-PEI Functionalized Mesoporous Silica Nanoparticles. Int. J. Nanomed. 2016, 11, 299–313. 10.2147/IJN.S94013. PMC4725644 26855569

[ref17] Elsayed A.; Al-Remawi M.; Maghrabi I.; Hamaidi M.; Jaber N. Development of Insulin Loaded Mesoporous Silica Injectable Particles Layered by Chitosan as a Controlled Release Delivery System. Int. J. Pharm. 2014, 461, 448–458. 10.1016/j.ijpharm.2013.12.014. 24368103

[ref18] Yellepeddi V. K.; Ghandehari H. Poly(Amido Amine) Dendrimers in Oral Delivery. Tissue Barriers 2016, 4, e1173773 10.1080/21688370.2016.1173773. 27358755 PMC4910834

[ref19] Wang B.; Zhang K.; Wang J.; Zhao R.; Zhang Q.; Kong X. Poly(Amidoamine)-Modified Mesoporous Silica Nanoparticles as a Mucoadhesive Drug Delivery System for Potential Bladder Cancer Therapy. Colloids Surf., B 2020, 189, 110832 10.1016/j.colsurfb.2020.110832. 32070865

[ref20] Grayson S. M.; Fréchet J. M. J. Convergent Dendrons and Dendrimers: From Synthesis to Applications. Chem. Rev. 2001, 101, 3819–3868. 10.1021/cr990116h. 11740922

[ref21] Fréchet J. M. J. Dendrimers and Supramolecular Chemistry. Proc. Natl. Acad. Sci. U.S.A. 2002, 99, 4782–4787. 10.1073/pnas.082013899. 11959930 PMC122668

[ref22] Radu D. R.; Lai C.-Y.; Jeftinija K.; Rowe E. W.; Jeftinija S.; Lin V. S. -Y. A Polyamidoamine Dendrimer-Capped Mesoporous Silica Nanosphere-Based Gene Transfection Reagent. J. Am. Chem. Soc. 2004, 126, 13216–13217. 10.1021/ja046275m. 15479063

[ref23] Janaszewska A.; Lazniewska J.; Trzepiński P.; Marcinkowska M.; Klajnert-Maculewicz B. Cytotoxicity of Dendrimers. Biomolecules 2019, 9, 330 10.3390/biom9080330. 31374911 PMC6723213

[ref24] Ghaferi M.; Koohi Moftakhari Esfahani M.; Raza A.; Al Harthi S.; Ebrahimi Shahmabadi H.; Alavi S. E. Mesoporous Silica Nanoparticles: Synthesis Methods and Their Therapeutic Use-Recent Advances. J. Drug Targeting 2021, 29, 131–154. 10.1080/1061186X.2020.1812614. 32815741

[ref25] Ficker M.; Paolucci V.; Christensen J. B. Improved Large-Scale Synthesis and Characterization of Small and Medium Generation PAMAM Dendrimers. Can. J. Chem. 2017, 95, 954–964. 10.1139/cjc-2017-0108.

[ref26] Šebestík J.; Reiniš M.; Ježek J. Synthesis of Dendrimers: Convergent and Divergent Approaches. In Biomedical Applications of Peptide-, Glyco- and Glycopeptide Dendrimers, and Analogous Dendrimeric Structures; Sebestik J.; Reinis M.; Jezek J., Eds.; Springer: Vienna, 2012; pp 55–81.

[ref27] Christensen J. B.; Pittelkow M.; Lewinsky R. Selective Synthesis of Carbamate Protected Polyamines Using Alkyl Phenyl Carbonates. Synthesis 2002, 15, 2195 10.1055/s-2002-34859.

[ref28] Cursaru B.; Teodorescu M.; Boscornea C.; Stanescu P. O.; Stoleriu S. Drug Absorption and Release Properties of Crosslinked Hydrogels Based on Diepoxy-Terminated Poly(Ethylene Glycol)s and Aliphatic Polyamines — a Study on the Effect of the Gel Molecular Structure. Mater. Sci. Eng. C 2013, 33, 1307–1314. 10.1016/j.msec.2012.12.030. 23827576

[ref29] Lee J. W.; Kim B.-K.; Kim H. J.; Han S. C.; Shin W. S.; Jin S.-H. Convergent Synthesis of Symmetrical and Unsymmetrical PAMAM Dendrimers. Macromolecules 2006, 39, 2418–2422. 10.1021/ma052526f.

[ref30] Li W.; Han Y.-C.; Zhang J.-L.; Wang B.-G. Effect of Ethanol on the Aggregation Properties of Cetyltrimethylammonium Bromide Surfactant. Colloid J. 2005, 67, 159–163. 10.1007/s10595-005-0075-7.

[ref31] Reviakine I.; Johannsmann D.; Richter R. P. Hearing What You Cannot See and Visualizing What You Hear: Interpreting Quartz Crystal Microbalance Data from Solvated Interfaces. Anal. Chem. 2011, 83, 8838–8848. 10.1021/ac201778h. 21939220

[ref32] Dixon M. C. Quartz Crystal Microbalance with Dissipation Monitoring: Enabling Real-Time Characterization of Biological Materials and Their Interactions. J. Biomol. Tech. 2008, 19, 151–158. 19137101 PMC2563918

[ref33] Wan F.; Herzberg M.; Huang Z.; Hassenkam T.; Nielsen H. M. A Free-Floating Mucin Layer to Investigate the Effect of the Local Microenvironment in Lungs on Mucin-Nanoparticle Interactions. Acta Biomater. 2020, 104, 115–123. 10.1016/j.actbio.2020.01.014. 31945503

[ref34] Feiler A. A.; Sahlholm A.; Sandberg T.; Caldwell K. D. Adsorption and Viscoelastic Properties of Fractionated Mucin (BSM) and Bovine Serum Albumin (BSA) Studied with Quartz Crystal Microbalance (QCM-D). J. Colloid Interface Sci. 2007, 315, 475–481. 10.1016/j.jcis.2007.07.029. 17706239

[ref35] Bravo-Osuna I.; Noiray M.; Briand E.; Woodward A. M.; Argüeso P.; Molina Martínez I. T.; Herrero-Vanrell R.; Ponchel G. Interfacial Interaction between Transmembrane Ocular Mucins and Adhesive Polymers and Dendrimers Analyzed by Surface Plasmon Resonance. Pharm. Res. 2012, 29, 2329–2340. 10.1007/s11095-012-0761-1. 22565639 PMC3867740

[ref36] Collado-González M.; González Espinosa Y.; Goycoolea F. M. Interaction Between Chitosan and Mucin: Fundamentals and Applications. Biomimetics 2019, 4, 32 10.3390/biomimetics4020032. 31105217 PMC6631199

[ref37] Freyer M. W.; Lewis E. A. Isothermal Titration Calorimetry: Experimental Design, Data Analysis, and Probing Macromolecule/Ligand Binding and Kinetic Interactions. In Methods in Cell Biology; Biophysical Tools for Biologists, Volume One: In Vitro Techniques; Academic Press, 2008; Vol. 84, pp 79–113. 10.1016/S0091-679X(07)84004-0 17964929

[ref38] Martín S. P.; Seeberger P. H.; Varón Silva D. Mucins and Pathogenic Mucin-Like Molecules Are Immunomodulators During Infection and Targets for Diagnostics and Vaccines. Front. Chem. 2019, 7, 710 10.3389/fchem.2019.00710. 31696111 PMC6817596

[ref39] Rose M. C. Mucins: Structure, Function, and Role in Pulmonary Diseases. Am. J. Physiol.: Lung Cell. Mol. Physiol. 1992, 263, L413–L429. 10.1152/ajplung.1992.263.4.L413. 1415719

[ref40] Menchicchi B.; Fuenzalida J. P.; Bobbili K. B.; Hensel A.; Swamy M. J.; Goycoolea F. M. Structure of Chitosan Determines Its Interactions with Mucin. Biomacromolecules 2014, 15, 3550–3558. 10.1021/bm5007954. 25122160

[ref41] Alahmadi S. M.; Mohamad S.; Maah M. J. Synthesis and Characterization of Mesoporous Silica Functionalized with Calix[]Arene Derivatives. Int. J. Mol. Sci. 2012, 13, 13726–13736. 10.3390/ijms131013726. 23202977 PMC3497351

[ref42] Date A. A.; Hanes J.; Ensign L. M. Nanoparticles for Oral Delivery: Design, Evaluation and State-of-the-Art. J. Controlled Release 2016, 240, 504–526. 10.1016/j.jconrel.2016.06.016. PMC5064878 27292178

[ref43] Braun K.; Stürzel C. M.; Kirchhoff F.; Lindén M. In Vitro Evaluation of a Peptide-Mesoporous Silica Nanoparticle Drug Release System against HIV-1. Inorganics 2020, 8, 42 10.3390/inorganics8070042.

[ref44] Björk E. M.; Atakan A.; Wu P.-H.; Bari A.; Pontremoli C.; Zheng K.; Giasafaki D.; Iviglia G.; Torre E.; Cassinelli C.; Morra M.; Steriotis T.; Charalambopoulou G.; Boccaccini A. R.; Fiorilli S.; Vitale-Brovarone C.; Robertsson F.; Odén M. A Shelf-Life Study of Silica- and Carbon-Based Mesoporous Materials. J. Ind. Eng. Chem. 2021, 101, 205–213. 10.1016/j.jiec.2021.06.011.

[ref45] Martínez Á.; Fuentes-Paniagua E.; Baeza A.; Sánchez-Nieves J.; Cicuéndez M.; Gómez R.; de la Mata F. J.; González B.; Vallet-Regí M. Mesoporous Silica Nanoparticles Decorated with Carbosilane Dendrons as New Non-Viral Oligonucleotide Delivery Carriers. Chem. – Eur. J. 2015, 21, 15651–15666. 10.1002/chem.201501966. 26361378

[ref46] Bansil R.; Turner B. S. The Biology of Mucus: Composition, Synthesis and Organization. Adv. Drug Delivery Rev. 2018, 124, 3–15. 10.1016/j.addr.2017.09.023. 28970050

[ref47] Park H.; Robinson J. R. Mechanisms of Mucoadhesion of Poly(Acrylic Acid) Hydrogels. Pharm. Res. 1987, 04, 457–464. 10.1023/A:1016467219657. 3508557

[ref48] Serra L.; Doménech J.; Peppas N. Engineering Design and Molecular Dynamics of Mucoadhesive Drug Delivery Systems as Targeting Agents. Eur. J. Pharm. Biopharm. 2009, 71, 519–528. 10.1016/j.ejpb.2008.09.022. 18976706 PMC2680154

[ref49] de Loubens C.; Lentle R. G.; Love R. J.; Hulls C.; Janssen P. W. M. Fluid Mechanical Consequences of Pendular Activity, Segmentation and Pyloric Outflow in the Proximal Duodenum of the Rat and the Guinea Pig. J. R. Soc. Interface 2013, 10, 20130027 10.1098/rsif.2013.0027. 23536539 PMC3645412

[ref50] Leal J.; Smyth H. D. C.; Ghosh D. Physicochemical Properties of Mucus and Their Impact on Transmucosal Drug Delivery. Int. J. Pharm. 2017, 532, 555–572. 10.1016/j.ijpharm.2017.09.018. 28917986 PMC5744044

[ref51] Seeliger E.; Lenhard D. C.; Persson P. B. Contrast Media Viscosity versus Osmolality in Kidney Injury: Lessons from Animal Studies. BioMed Res. Int. 2014, 2014, 358136 10.1155/2014/358136. 24707482 PMC3950904

[ref52] Salis A.; Parsons D. F.; Boström M.; Medda L.; Barse B.; Ninham B. W.; Monduzzi M. Ion Specific Surface Charge Density of SBA-15 Mesoporous Silica. Langmuir 2010, 26, 2484–2490. 10.1021/la902721a. 19831379

[ref53] Liu Y.; Bryantsev V. S.; Diallo M. S.; Goddard W. A. III PAMAM Dendrimers Undergo PH Responsive Conformational Changes without Swelling. J. Am. Chem. Soc. 2009, 131, 2798–2799. 10.1021/ja8100227. 19199433

[ref54] Mortensen J. S.; Saaby L.; Harloff-Helleberg S.; Mørck Nielsen H. Barrier Properties of Ex Vivo Porcine Intestinal Mucus Are Highly Independent of Isolation and Storage Conditions. Eur. J. Pharm. Biopharm. 2022, 16, 106–110. 10.1016/j.ejpb.2022.03.015. 35364256

