# Supplementary material for: Mucoadhesive Dendrons Conjugated to Mesoporous Silica Nanoparticles as a Drug Delivery Approach for Orally Administered Biopharmaceuticals
Source: ACS Appl Mater Interfaces. 2023 Feb 7;15(7):8798–810. doi: 10.1021/acsami.2c16502 (PMC9951175; doi:10.1021/acsami.2c16502)
Supplement: Supplementary file 1 — am2c16502_si_001.pdf [file am2c16502_si_001.pdf]

Supporting Information

Mucoadhesive dendrons conjugated to mesoporous silica nanoparticles as a drug delivery approach for orally administered biopharmaceuticals

*Matteo Tollemeto[a, b, d], Zheng Huang[b], Jørn B. Christensen[a], Hanne Mørck Nielsen[b] & Stine Rønholt[b,c *]*

[a] Department of Chemistry, Thovaldsensvej 40, DK-1871 Frederiksberg, Denmark

[b] Center for Biopharmaceuticals and Biobarriers in Drug Delivery (BioDelivery), Department of Pharmacy, Faculty of Health and Medical Sciences, University of Copenhagen, Universitetsparken 2, 2100 Copenhagen Ø, Denmark

[c] Current affiliation: LEO Foundation Center for Cutaneous Drug Delivery, Department of Pharmacy, Faculty of Health and Medical Sciences, University of Copenhagen, Universitetsparken 2, 2100 Copenhagen Ø, Denmark

[d] Current affiliation: Department of Health Technology, DTU Health Tech, Technical University of Denmark, 2800 Kgs., Lyngby, Denmark

* Corresponding author: stine.roenholt@sund.ku.dk

NMR

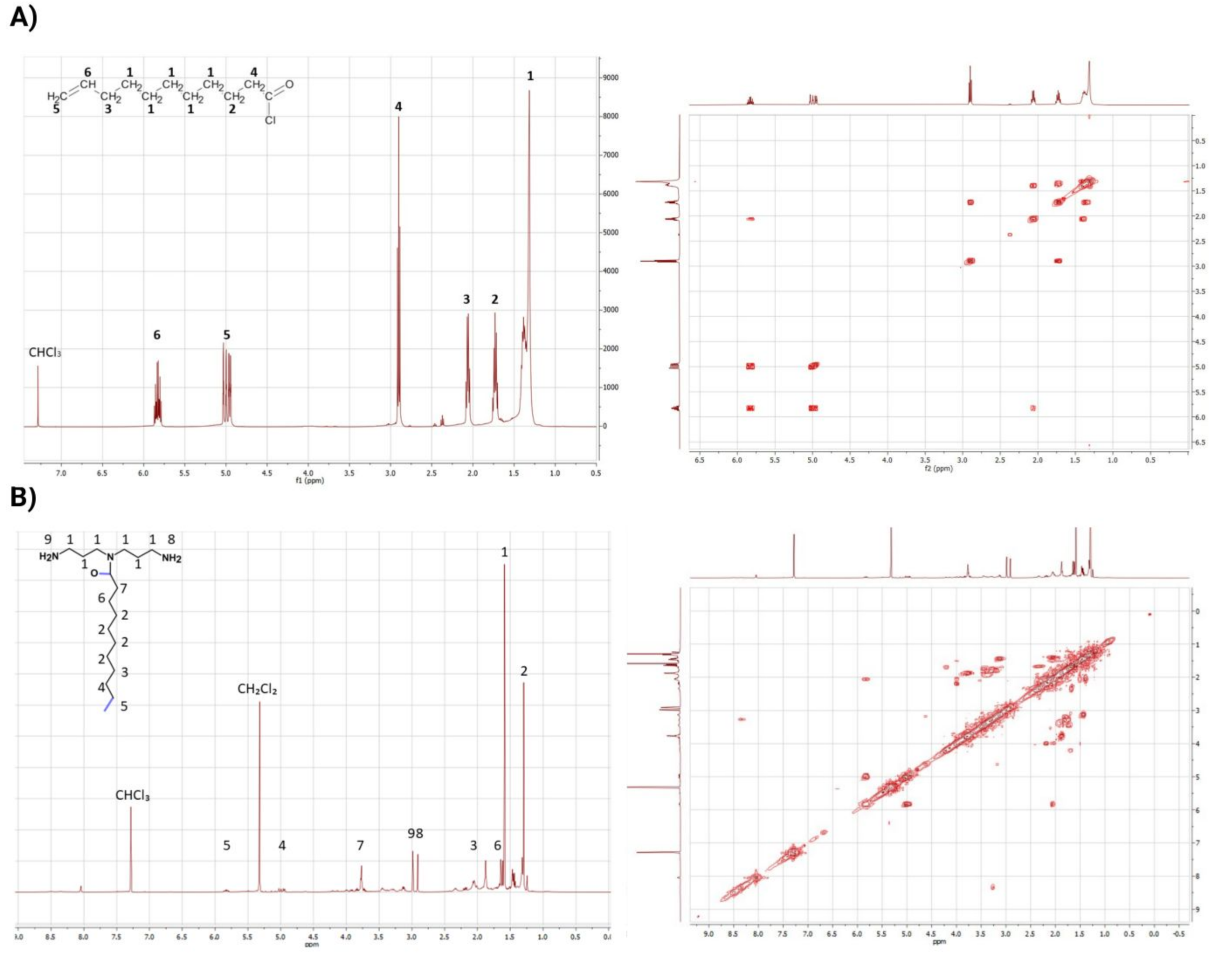

Figure S1| NMR spectra. A) ^{1}H-NMR (300 MHz, $CDCl_3$) spectra of 10-undecenoyl chloride; two-dimensional ^{1}H-^{1}H COSY spectrum of 10-undecenoyl chloride. B) ^{1}H-NMR (300 MHz, $CDCl_3$) spectra of G1 dendrons; two-dimensional ^{1}H-^{1}H COSY spectrum of G1 dendrons.

MS

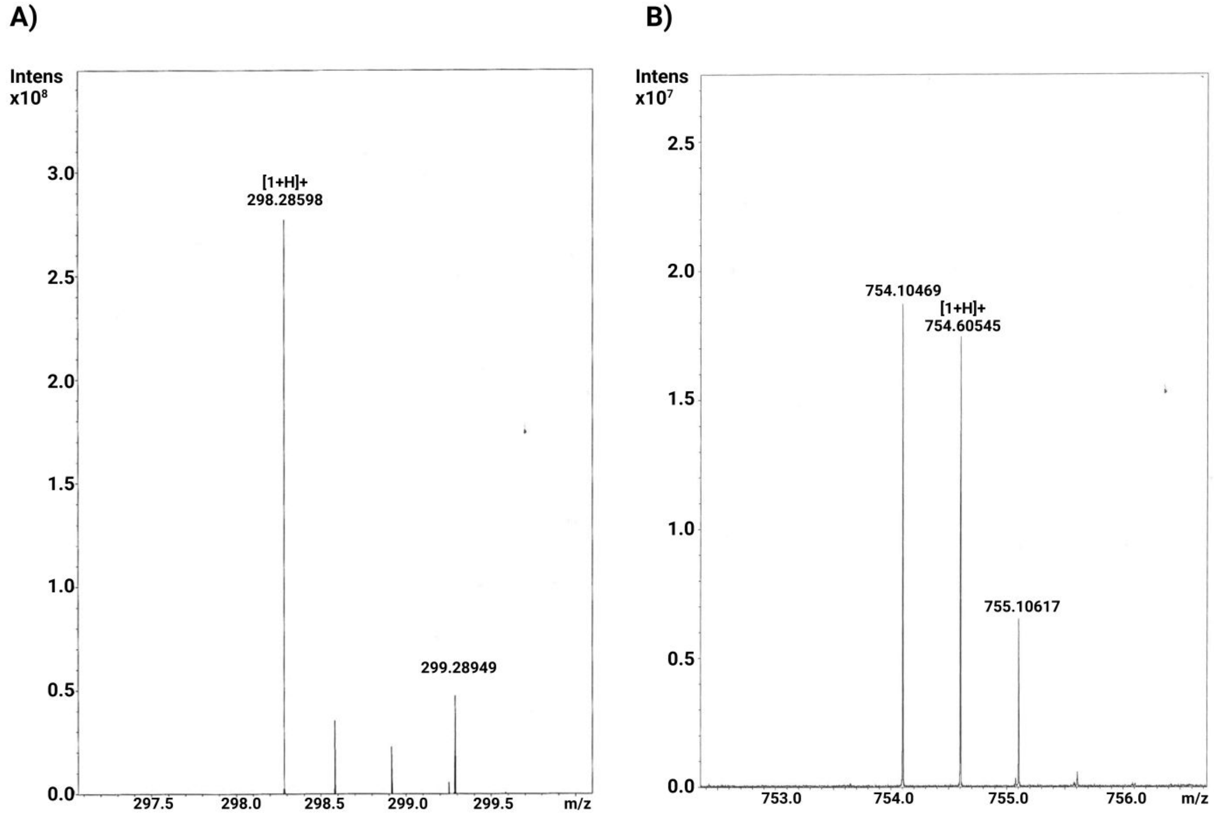

Figure S2| Mass spectra of G1 and G2 dendrons. A) Mass spectrum of a solution of G1 dendrons in MeOH, showing the mass of a protonated dendron ions [1+H]+ at 299.28 m/z; B) mass spectrum of a solution of G2 dendrons in MeOH, showing the mass of a protonated dendron ions [1+H]+ at 754.60 m/z.

QCM-D

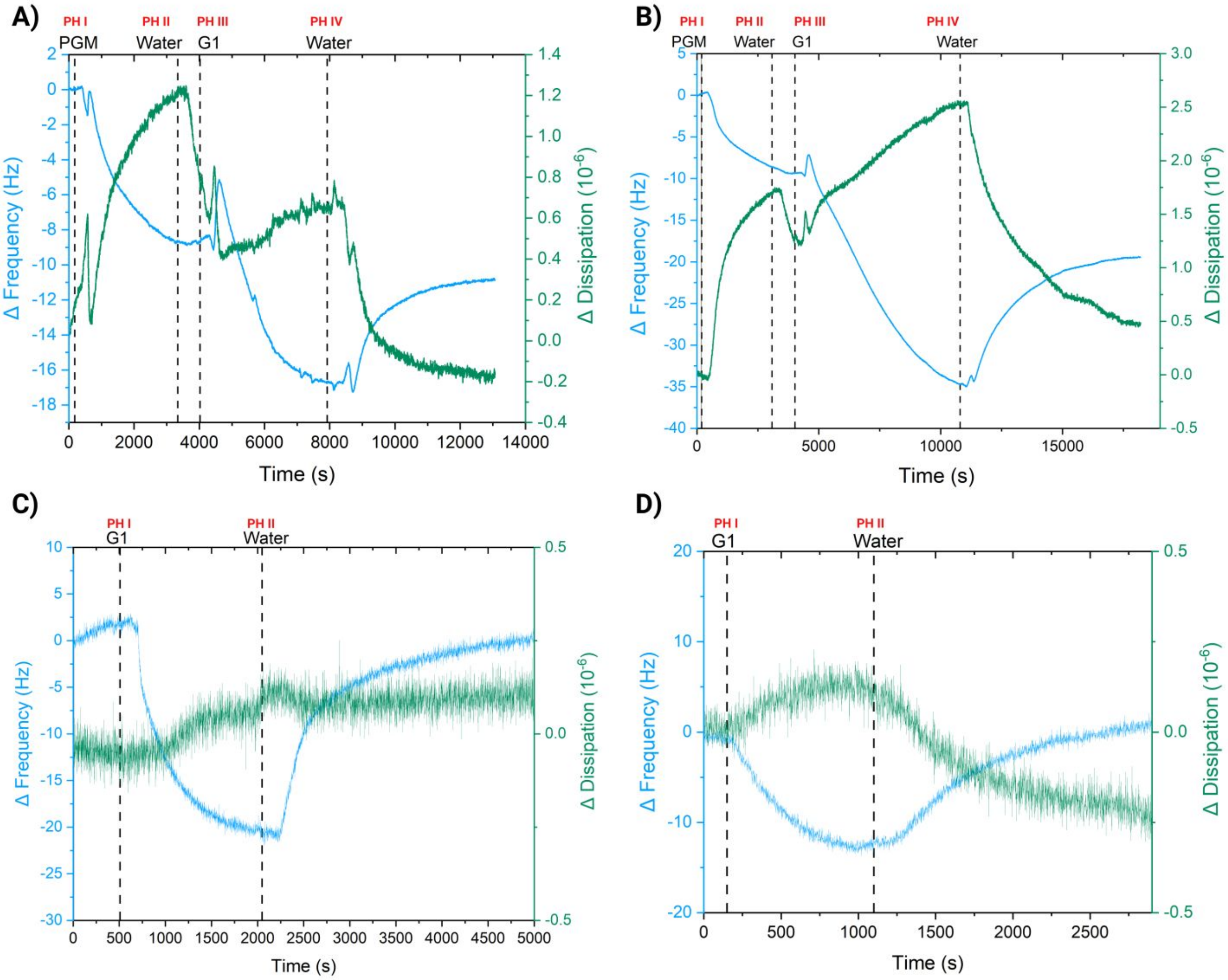

Figure S3| Quartz crystal microbalance with dissipation (QCM-D) measurements. A and B) Show two additional representations of quartz crystal microbalance with dissipation (QCM-D) results displaying the interactions of G1 dendrons with a layer of mucin conjugated to the gold surface as illustrated. The graph shows the interaction between mucin layer and the generation 1 (G1) dendrons in pH 6.5 ultrapure water at 25 ˚C through the change in frequency (Δf) in blue and dissipation (ΔD) in green for the fifth overtone as a function of time. Phase I: The formation of physically adsorbed mucin layer. Phase II: Rinsing with ultrapure water. Phase III: Addition

of G1 dendrons. Phase IV: Rinsing with ultrapure water; C and D) Show two control experiment representations of QCM-D results displaying the adsorption of G1 dendrons to the gold surface and washing off the layer. The graph shows the interaction between the G1 dendrons in pH 6.5 ultrapure water at 25 ˚C through the change in frequency (Δf) in blue and dissipation (ΔD) in green for the fifth overtone as a function of time. Phase I: The formation of a physically adsorbed dendrons layer. Phase II: Rinsing with ultrapure water.

ITC

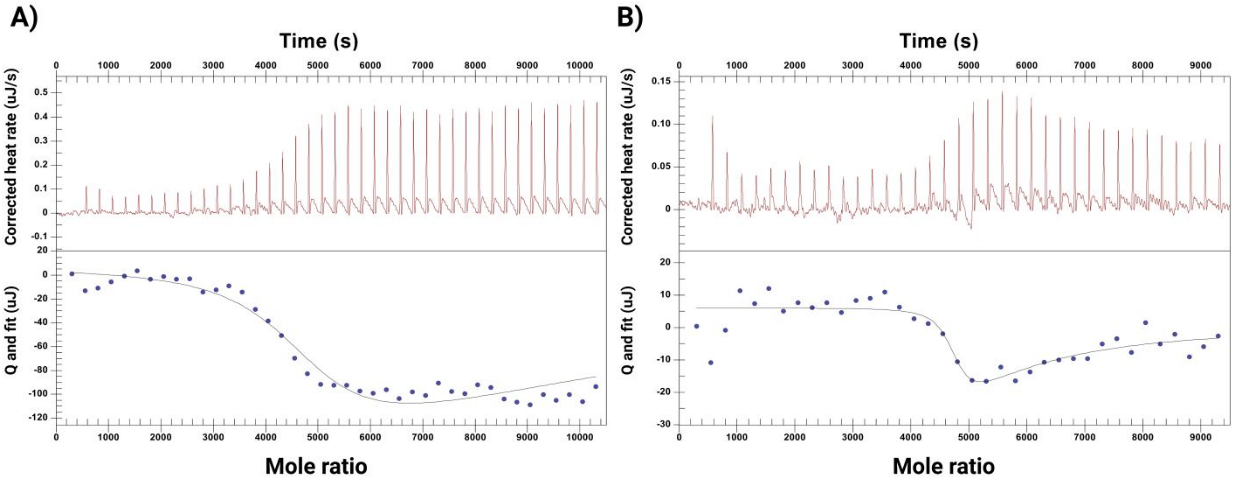

Figure S4| Isothermal calorimetry (ITC) titration measurements. A and B) Show two additional replicates for ITC titration profile of raw heats of binding (top panel) for mucin (0.3 mg/mL) titrated with generation 1 (G1) dendrons (0.052 mM) in pH 6.5 ultrapure water at 25 ˚C. The lower panel shows a fit of the integrated heats of binding with a multiple sites binding curve, excluding dilution effects using the NanoAnalyze software.

Surface charge

MeSiNP:Dendrimers ratio (w:w)	ζ-potential ± SD in mV

1:1	-40.4 ± 3.16
1:5	-29.0 ± 1.08
1:20	-28.5 ± -2.23

Table S1| Conjugation and characterization of generation 1 dendrons on mesoporous silica nanoparticles. The table shows the ζ-potential for MeSiNP and G1_MeSiNP for different conjugation ratios (w:w).

Rheology

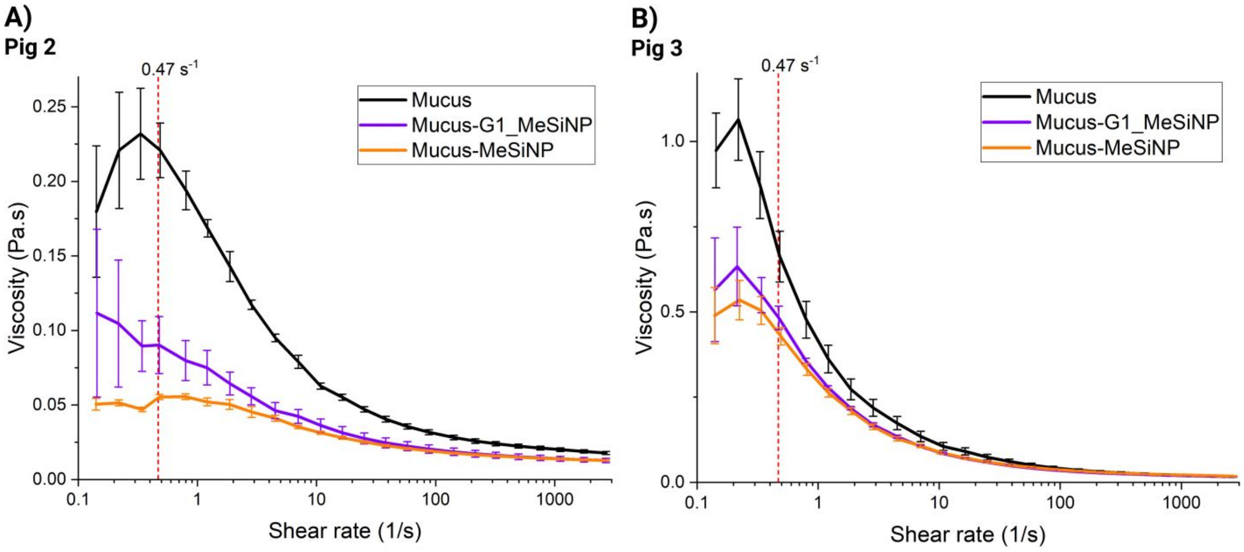

Figure S5| Rheological measurements of nanoparticles interacting with mucus. A and B) Show viscosity values in the mucus (black) samples from pig 2 and 3, mucus-MeSiNP (orange) and mucus-G1_MeSiNP (purple). Results are shown as average ± SD, (n=3).
